# Single-nucleus RNA-seq identifies divergent populations of FSHD2 myotube nuclei

**DOI:** 10.1371/journal.pgen.1008754

**Published:** 2020-05-04

**Authors:** Shan Jiang, Katherine Williams, Xiangduo Kong, Weihua Zeng, Nam Viet Nguyen, Xinyi Ma, Rabi Tawil, Kyoko Yokomori, Ali Mortazavi

**Affiliations:** 1 Department of Developmental and Cell Biology, University of California Irvine, Irvine, California, United States of America; 2 Center for Complex Biological Systems, University of California Irvine, Irvine, California, United States of America; 3 Department of Biological Chemistry, School of Medicine, University of California Irvine, Irvine, California, United States of America; 4 Neuromuscular Disease Unit, Department of Neurology, University of Rochester Medical Center, Rochester, New York, United States of America; Helmholtz Zentrum Munchen Deutsches Forschungszentrum fur Umwelt und Gesundheit, GERMANY

## Abstract

FSHD is characterized by the misexpression of *DUX4* in skeletal muscle. Although *DUX4* upregulation is thought to be the pathogenic cause of FSHD, *DUX4* is lowly expressed in patient samples, and analysis of the consequences of *DUX4* expression has largely relied on artificial overexpression. To better understand the native expression profile of *DUX4* and its targets, we performed bulk RNA-seq on a 6-day differentiation time-course in primary FSHD2 patient myoblasts. We identify a set of 54 genes upregulated in FSHD2 cells, termed FSHD-induced genes. Using single-cell and single-nucleus RNA-seq on myoblasts and differentiated myotubes, respectively, we captured, for the first time, *DUX4* expressed at the single-nucleus level in a native state. We identified two populations of FSHD myotube nuclei based on low or high enrichment of *DUX4* and FSHD-induced genes (“FSHD-Lo” and “FSHD Hi”, respectively). FSHD-Hi myotube nuclei coexpress multiple DUX4 target genes including *DUXA*, *LEUTX* and *ZSCAN4*, and also upregulate cell cycle-related genes with significant enrichment of E2F target genes and p53 signaling activation. We found more FSHD-Hi nuclei than *DUX4*-positive nuclei, and confirmed with *in situ* RNA/protein detection that *DUX4* transcribed in only one or two nuclei is sufficient for DUX4 protein to activate target genes across multiple nuclei within the same myotube. *DUXA* (the *DUX4* paralog) is more widely expressed than *DUX4*, and depletion of *DUXA* suppressed the expression of *LEUTX* and *ZSCAN4* in late, but not early, differentiation. The results suggest that the DUXA can take over the role of DUX4 to maintain target gene expression. These results provide a possible explanation as to why it is easier to detect DUX4 target genes than *DUX4* itself in patient cells and raise the possibility of a self-sustaining network of gene dysregulation triggered by the limited *DUX4* expression.

## Author summary

Although misexpression of *DUX4* has been known as the major cause in FSHD, it is lowly expressed in patient samples and analysis of the consequences of *DUX4* expression has largely relied on artificial overexpression. Here, we took advantage of recent methodological advances to observe native *DUX4* expression at the single-nucleus level in FSHD2 patient-derived myotubes. Using single-nucleus RNA-seq (snRNA-seq), we were able to detect endogenous *DUX4*-expressing nuclei and the extent of spreading of DUX4-target gene expression across many nuclei. Our highly sensitive snRNA-seq method further allowed us to identify two populations of FSHD myotube nuclei with distinct transcriptional profiles. One is highly enriched with *DUX4* and target genes (FSHD-Hi) while the other has sparser *DUX4* and FSHD-induced genes expressed (FSHD-Lo), reflecting two potentially different pathological states of patient myotubes. We observed a set of transcription factors specifically upregulated in FSHD-Hi myotube nuclei associated with the cell cycle, and significant upregulation of DUX4 paralog *DUXA* that contributes to further upregulation of DUX4 target genes. We propose that transcription factors downstream of DUX4 may amplify DUX4 signal and thus act to perpetuate FSHD.

## Introduction

Facioscapulohumeral muscular dystrophy (FSHD) is one of the most common inherited muscular dystrophies and is characterized by progressive wasting of facial, shoulder and upper arm musculature [1]. The most common form of FSHD, FSHD1 (>95% of cases), is linked to the mono-allelic contraction of the D4Z4 macrosatellite repeat array on chromosome 4q from 11–100 units to 1–10 units, with each 3.3 kb repeat containing the open reading frame for the double-homeobox transcription factor DUX4 [2–4]. In contrast, FSHD2 (<5% of FSHD cases) has no contraction of the chromosome 4q repeat array. Approximately 80% of FSHD2 cases are characterized by recurring mutations in the chromatin modifier SMCHD1 (Structural Maintenance of Chromosomes flexible Hinge Domain-containing protein 1) on chromosome 18 [5]. SMCHD1 is important for maintenance of DNA methylation and epigenetic silencing of multiple genomic loci, including the D4Z4 repeat array [5]. Studies have also found that SMCHD1 mutations can act as disease modifiers in severe cases of FSHD1 [6, 7].

FSHD is associated with the expression of the full-length *DUX4* transcript (*DUX4fl*) which is stabilized by a specific single-nucleotide polymorphism in the chromosomal region distal to the last D4Z4 repeat creating a canonical polyadenylation signal [8–10]. *DUX4fl* encodes a transcriptional activator with a double-homeobox domain that binds to a specific sequence motif upstream of its target genes in the genome [3, 4]. Normal expression of *DUX4* is restricted to brief expression in 4-cell human embryos when it activates genes for zygote genome activation (ZGA), and in the testis [11–13]. In muscle cells, overexpression of *DUX4fl* causes differentiation defects and cytotoxicity in human and mouse myoblasts [14, 15]. However, the endogenous *DUX4fl* is expressed at extremely low levels in FSHD and DUX4 protein is only detected in 0.1% and 0.5% of patient myoblasts and myotubes, respectively, *in vitro* [16]. The relationship of DUX4-positive and -negative cells and whether DUX4-negative patient cells contribute to the disease is unclear. The regulation of *DUX4* expression is controlled by multiple epigenetic processes. D4Z4 repeats are normally heterochromatic with DNA hypermethylation and histone H3 lysine 9 trimethylation (H3K9me3), which are significantly reduced in FSHD1 and FSHD2 [17, 18]. The depletion of SMCHD1, which binds to D4Z4 repeats in an H3K9me3-dependent fashion [2], results in *DUX4fl* upregulation and mutations throughout the gene correlate with CpG hypomethylation in D4Z4 repeats [19].

Here we focused on the *SMCHD1*-mutated FSHD2 subtype in order to characterize the heterogeneity of *DUX4* and FSHD-induced target gene expression at the single-cell level using *in vitro* differentiation of primary FSHD2 patient-derived myoblasts into myotubes. Although FSHD2 represents a minor population of FSHD cases, patient cells exhibit comparable clinical and gene expression phenotype as FSHD1 [20]. We used two FSHD2 patient samples with defined genetic mutations of *SMCHD1* and significant DNA hypomethylation of D4Z4 (S1 Table). Using bulk RNA-seq, we profiled gene expression patterns during a differentiation time-course and identified candidate disease-related key genes (i.e. FSHD-induced genes) that are upregulated specifically in FSHD cells by comparing expression profiles between FSHD2 and control. We then used single-cell RNA-seq in myoblasts and single-nucleus RNA-seq [21] in day 3 and day 5 post-differentiation myotubes to characterize the expression patterns of *DUX4* and other FSHD-induced genes. We sucessfully detected the first set of single nuclei with endogenous *DUX4* expression (*DUX4*-detected) from FSHD myotubes. We found that *DUX4* transcript-positive nuclei do not necessarily co-express all the FSHD-induced genes whereas a much larger set of FSHD myotube nuclei express multiple FSHD-induced genes. We performed cluster analyses and identified multiple subpopulation of FSHD nuclei with distinct gene expression signatures. In particular, we found that FSHD nuclei can be subcategorized into two populations based on high or low FSHD-induced gene expression levels (termed FSHD-Hi and FSHD-Lo, respectively). Further analyses of these two populations revealed expression of distinct sets of transcription factors related to cell cycle regulation in the FSHD-Hi nuclei, indicating their distinct cellular states. Interestingly, we found that the DUX4 target and paralog, DUXA, is widely expressed and maintains other DUX4 target gene expression, which may provide insight into how rare expression of *DUX4* results in a wide-spread dystrophic phenotype.

## Results

### Upregulation of FSHD-induced genes during FSHD2 myotube differentiation

**Fig 1:**
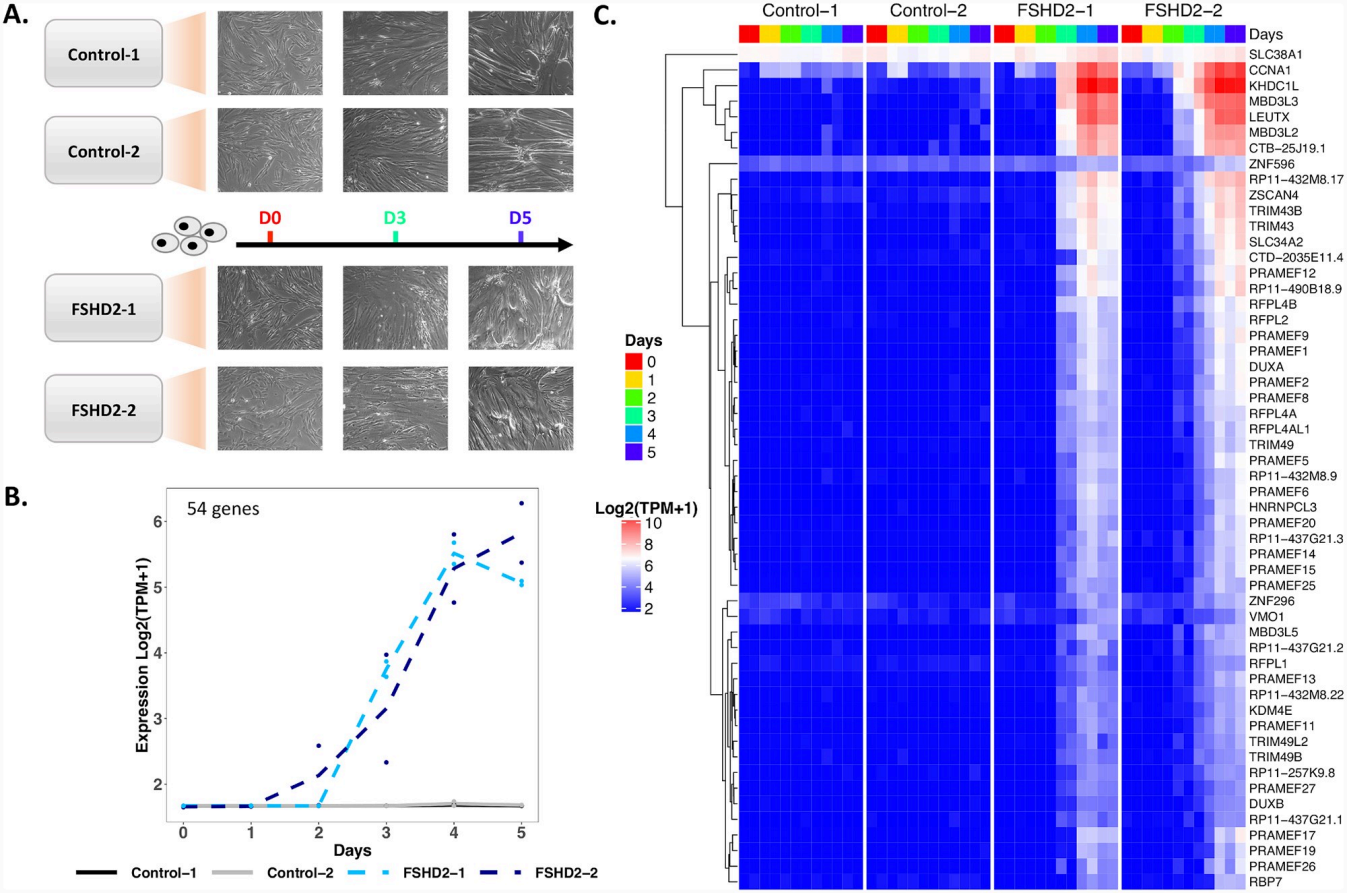
Upregulation of FSHD-induced genes starting at day 2 identified in bulk RNA-seq time-course. **(A)** Differentiation time-course of control and FSHD2 patient-derived myoblasts to myotubes. Morphology changes are shown for days 0, 3 and 5 of differentiation. **(B)** Average expression profile of 54 genes upregulated in FSHD2 cells starting at day 2 of differentiation. maSigPro clustered 10,827 genes into three clusters based on their expression patterns during control and FSHD2 differentiation time-course. **(C)** Hierarchical heatmap of gene expression values of the 54 genes from (B). Expression values in transcripts per million (TPM) are TMM and log normalized. We refer to these 54 genes and *DUX4* as “FSHD-induced genes”.

Previous studies indicated that *DUX4* is upregulated during FSHD patient myoblast differentiation [22]. In order to understand the temporal expression differences between FSHD2 patient-derived and control myoblasts, we differentiated these *in vitro* to measure the dynamics of gene expression in a 6-day time-course using conventional bulk RNA sequencing (RNA-seq) (Fig 1A and S1 Fig) (Methods). We used two independent primary control myoblast samples from tibialis anterior, Control-1 and Control-2, and two from quadricep, Control-3 and Control-4, and two independent primary FSHD2 myoblast samples from tibialis anterior, FSHD2-1 and FSHD2-2, which have known *SMCHD1* mutations (S1 Table). After sequencing two biological replicate RNA samples for each of the six cell lines every day for six days, we filtered out lowly expressed genes and kept 10,827 genes for downstream analysis. We do not detect *DUX4* from the RNA-seq probably due to few nuceli expressing *DUX4*, but we detect the induction of *DUX4-fl* via RT-qPCR (S2 Fig). We looked for differences between the control and FSHD2 myoblasts from the tibialis anterior using principal component analysis (PCA) (S3 Fig) and for all the samples (S4 Fig). We observed that the days of differentiation aligned to each other across cell lines following a clear trajectory of myogenesis (PC1, 51.9% variance in expression; PC2, 13.2% variance in expression). We also found that the two FSHD2 cell lines diverge from the two tibialis anterior control cell lines for days 3 to 5 in two principal components with known genes upregulated in FSHD driving the variance (PC3, 5.9% variance in expression; PC4, 4.0% variance in expression) (S1 Fig, S2 Table). Thus, FSHD2 patient-derived myotubes can be distinguished from control cells by day 3 of differentiation when profiling transcriptomes at the population level.

In order to identify temporal patterns of expression, we used maSigPro [23] to cluster genes into three clusters based on expression over time (Fig 1B and S5 Fig) (Methods). A set of 54 genes are specifically upregulated in FSHD2 starting at day 2 (Cluster 3) (Fig 1B and 1C). We define these 54 upregulated genes along with *DUX4* as “FSHD-induced genes” (Fig 1B and 1C). Genes in this cluster were highly enriched in GO terms for negative regulation of cell differentiation (p = 1x10^-12.9^) and methylation-dependent chromatin silencing (p = 1x10^-7.17^) (S3 Table). Of these 54 genes, 53 were previously identified as possible DUX4 targets from myoblasts with inducible DUX4 [24], endogenous DUX4 [22] or FSHD biopsies [20] (S6 Fig). While these genes overlap with those upregulated in response to *DUX4* expression, they may not be direct DUX4 target genes since DUX4 turns on other transcriptional regulators. For this reason we refer to these as “FSHD-induced genes”. These genes were upregulated in waves starting at day 2, such as *LEUTX* and *ZSCAN4*, followed by day 3, such as *CCNA1* and *DUXA*, and day 4, such as *DUXB* (Fig 1C and S7 Fig). After being significantly upregulated, most FSHD-induced genes remained upregulated through the end of the time-course, including two DUX4 paralogs, *DUXA* and *DUXB* (Fig 1C and S7 Fig) [25].

The other two clusters of genes identified from maSigPro represent genes increasing (Cluster 2) or decreasing (Cluster 1) in expression in both FSHD2 and control across the timecourse (S5 Fig, S3 Table). GO terms for these clusters include muscle system process (p = 1x10^-67.0^) and muscle structure development (p = 1x10^-47.1^) for cluster 2, and RNA splicing (p = 1x10^-11.5^) for cluster 1 (S3 Table). Myogenesis genes, such as ACTA1 and MYOG, are in cluster 2. Both FSHD2 and control samples have similar expression levels in both these clusters across time (S5A and S5B Fig), suggesting that the control and FSHD2 samples seem to differentiated at similar efficiencies. We also monitored the differentiation of Control-2 and FSHD2-2 by differentiation index and MYH1 staining (S5C Fig). The differentiation index of FSHD2-2 is statistically lower than that of Control-2 at day 3, but the two are not statistically different by day 5. Altered myogenesis in FSHD cells has been shown in previous studies [26]. Recently, a study showed upregulation and incorporation of alternate histones H3.X and H3.Y following DUX4 expression [27]. In this study, *H3*.*Y* (AKA *RP11-432M8*.*17*) has increased expression in FSHD2 cells and is included in our FSHD-induced genes. *H3*.*X (RP11-321E2*.*13)* is classified as a pseudogene in the reference we use and was therefore not included in our analysis. In summary, we found a set of genes significantly upregulated in differentiating FSHD2 myotubes by day 3 which we term FSHD-induced genes along with *DUX4*.

### Detection of nuclei with *DUX4* expression from FSHD2 myotubes using single-nucleus full-length RNA-seq

Although we failed to detect *DUX4* in our bulk RNA-seq, the upregulation of FSHD-induced genes was nevertheless observed during myotube differentiation specifically in FSHD2 samples. We wondered whether the expression of FSHD-induced genes is seen in every cell and whether the expression of *DUX4* and DUX4-target genes were indeed present only in a subset of cells. We therefore performed single-cell RNA-seq on undifferentiated myoblasts and single-nucleus RNA-seq on myotubes using the Smart-Seq protocol on the Fluidigm C1 platform [21] at day 3 of differentiation using control and FSHD2 primary cells (S8A Fig). Day 3 was chosen as it was the first day of robust FSHD-induced gene expression in the differentiation time-course thereby allowing us to observe early transcriptional changes. Additionally, we selected FSHD2-2 based on the higher expression level of FSHD-induced genes compared to FSHD2-1 during differentiation (Fig 1B and 1C). The Fluidigm C1 platform enables us to prepare full-length cDNA libraries from up to 96 cells or nuclei at a time. We captured a total of 317 cells and nuclei with an average read depth of 2,624,274 per cell or nucleus and kept cells and nuclei with at least 500 genes detected (S8A Fig). As quality control that our single cell data matched our bulk time-course, we first pooled reads from all single cells/single nuclei for each cell type and performed incremental PCA with the bulk time-course RNA-seq samples for these cell lines (S8B and S8C Fig). As expected, the pooled single cell myoblasts clustered with day 0 samples in both control and FSHD2. For the pooled myotube single nuclei, FSHD2 replicate 1 (FSHD2 R1) aligned with day 3 of the FSHD2 time-course, but FSHD2 replicate 2 (FSHD2 R2) located between control and FSHD2 day 3 in the time-course (S8C Fig). This suggests variable differentiation efficiencies for the two replicates, which could be caused by subtle differences in seeding density.

**Fig 2:**
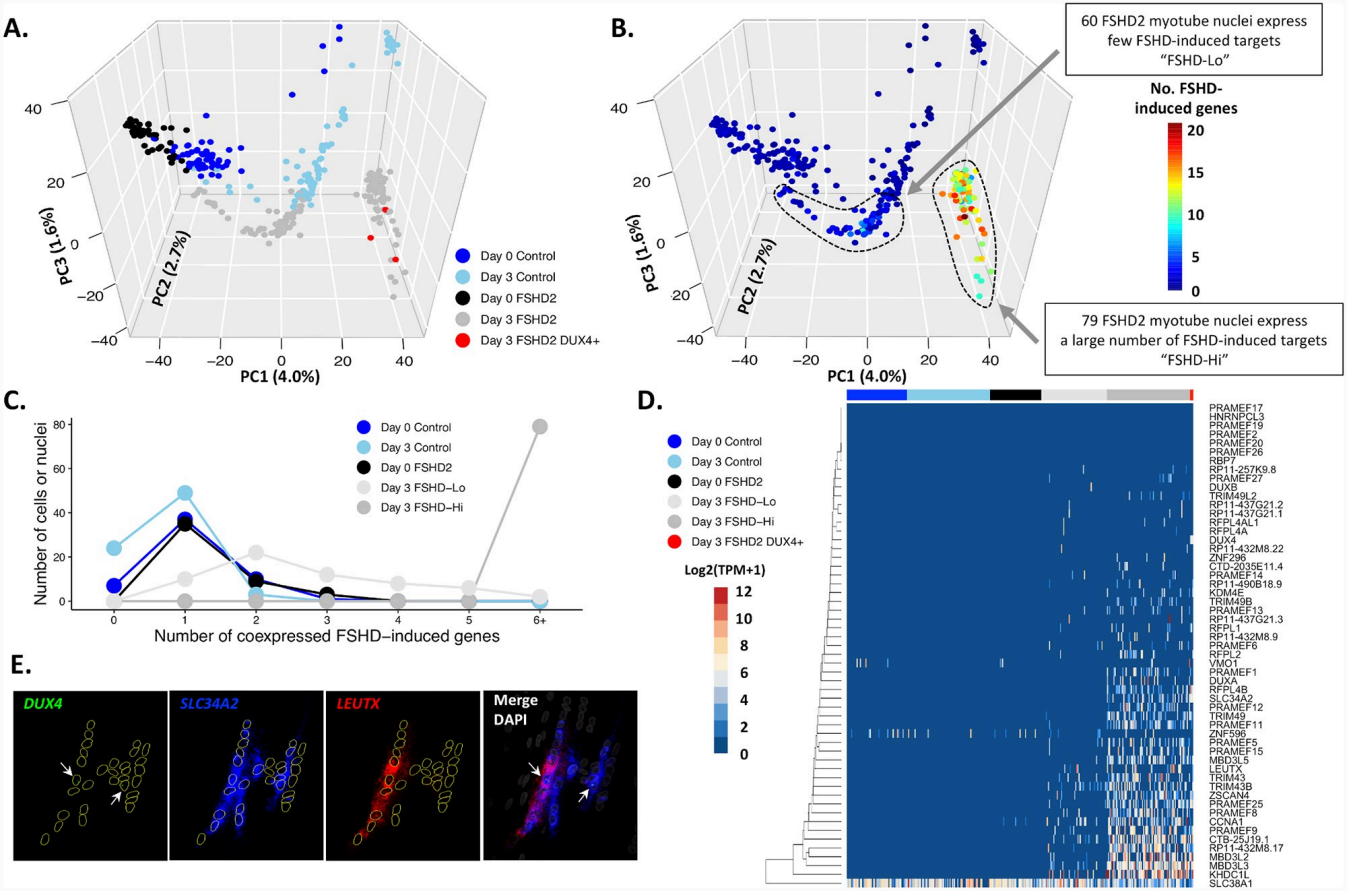
FSHD2 myotube nuclei can be separated into two clusters with differential expression of FSHD-induced genes. **(A)** PCA of single-cell (for myoblast) and single-nucleus (for myotube) RNA-seq data for control-3 and FSHD2-2. Cell types are labeled by color, and three DUX4-detected FSHD2 myotube nuclei are specifically labeled in red. **(B)** PCA from panel (A) colored by the number of FSHD-induced genes detected (TPM >1) defined in Fig 1. **(C)** Summary of the number of FSHD-induced genes coexpressed (TPM >0) in different cell types. Cell lines and days are labeled by color. **(D)** Heatmap of the expression of FSHD-induced genes in single-cell myoblasts and single-nuclei from myotubes. The bar is colored by cell line and day. **(E)** RNA FISH (RNAScope) of *DUX4*, *LEUTX* and *SLC34A2* in FSHD2 myotubes at day 3 of differentiation. *DUX4*, green; *LEUTX*, red; *SLC34A2*, blue; DAPI, white. Arrow indicate *DUX4* spots in green. We examined 240 myotubes, of which 11 myotubes were found to be *DUX4*-positive and 7 of them co-expressed both *LEUTX* and *SLC34A2* while 2 co-expressed *SLC34A2* only. Two additional myotubes expressed *LEUTX*/*SLC34A2* without detectable *DUX4* signal, and 4 appear to express *SLC34A2* only.

Importantly, we found that 3 out of 79 (3.8%) nuclei in FSHD2 R1 showed high expression of *DUX4* (11.24 TPM, 34.15 TPM and 68.49 TPM) while we found no *DUX4*-detected nuclei in FSHD2 R2, revealing the high level of heterogeneity in the FSHD2 cell population with *DUX4* only expressed in a small fraction of nuclei. We then analyzed the global profiles of the single-cell and single-nucleus transcriptomes using PCA analysis and found that all 3 *DUX4*-detected nuclei as well as other FSHD2 R1 nuclei clearly separated from FSHD2 R2 and control myotube nuclei (Fig 2A). Co-clustering of both *DUX4*-positive and negative nuclei of FSHD2 R1 suggests that they might come from the same myotubes as cell fusion was not blocked during differentiation in our study. Diffusion of the DUX4 protein to multiple nuclei was demonstrated previously despite *DUX4* mRNA transcription in only a few nuclei of the same myotube [22]. We further confirm this by RNA-protein costaining of DUX4 (S11B Fig). We analyzed the 55 genes, which includes *DUX4* and FSHD-induced genes, genes specifically upregulated at day 3 or later during our bulk time-course of FSHD2 differentiation (Fig 1B and 1C), and observed that these genes showed significant enrichment in FSHD2 R1 myotube nuclei compared with control myotube nuclei (p<2e-16). Nuclei with the highest enrichment clustered with the 3 *DUX4*-detected nuclei, and thus we labeled this group of nuclei “FSHD-induced genes high” (FSHD-Hi) (Fig 2B and S9 Fig). The FSHD2 R2 myotube nuclei also showed significantly higher enrichment of FSHD-induced genes than control myotube nuclei (p<2e-16) but had fewer FSHD-induced genes expressed than the FSHD-Hi group, and therefore this group of nuclei was labeled “FSHD-induced genes low” (FSHD-Lo) (Fig 2B and S9 Fig). We found that all myoblast cells and control myotube nuclei rarely express more than 2 FSHD-induced genes (Fig 2C), whereas FSHD-Lo nuclei coexpress between 1 to 6 and at most 9 of the FSHD-induced genes. However, all FSHD-Hi nuclei express at least 6 of these genes with most coexpressing at 12 and up to 22 genes (Fig 2C). In summary, we detected two different patient myotube nuclei populations: (1) a set of 79 nuclei that express FSHD-induced genes (FSHD-Hi), 3 of which express endogenous *DUX4* (*DUX4*+); (2) 60 nuclei that are clearly different from control nuclei but with no *DUX4* detected and significantly lower FSHD-induced gene expression (FSHD-Lo).

Interestingly, we observed the expression of DUX4 paralogs *DUXA* and *DUXB* expressed in FSHD2 myotube nuclei. *DUXA* was expressed exclusively in the FSHD-Hi nuclei population. We found that 34 FSHD-induced genes were expressed in both FSHD-Hi and FSHD-Lo populations, including reported DUX4 targets *LEUTX*, *ZSCAN4*, *MBD3L2*, *TRIM43*, *KHDC1L* and *CCNA1* [4, 20, 25] indicating that they may perform as a core set of responsive and interactive genes during FSHD progression (Fig 2D). We observed that FSHD-Hi and FSHD-Lo have distinct coexpression patterns which indicates different cell states. Within the FSHD-Hi nuclei, a large number of the FSHD-induced genes are coexpressed with transcription factors, such as *LEUTX* and *DUXA*, but not *DUX4* (Fig 2D). Taken together, two identified patient myotube nuclei populations, FSHD-Hi with a small set of *DUX4*-detected nuclei and FSHD-Lo, exhibit distinct co-expression patterns of FSHD-induced genes including DUX4-target transcription factor genes.

To assess whether these groups of nuclei have distinct expression of FSHD-induced genes, we determined the coexpression patterns between a subset of FSHD-induced genes which had variable expression in the single cells and nuclei. To determine expression profiles of *DUX4*-detected nuclei, we examined genes coexpressed with *DUX4*. We found that *DUX4* was coexpressed with 23 FSHD-induced genes including two transcription factors, *LEUTX* and *ZSCAN4*, which have been reported as DUX4 targets in FSHD (S6 and S10 Figs) [22, 24]. *DUX4* and *ZSCAN4* were expressed in all three *DUX4*-detected nuclei while *DUX4* and *LEUTX* were only simultaneously expressed in one *DUX4*-detected nuclei. FSHD-induced genes coexpressed in all three *DUX4*-detected nuclei include *KHDC1L*, *PRAMEF25*, *PRAMEF9*, *RFPL4B*, *RP11-432M8*.*17*, *SLC34A2*, *SLC38A1* and *ZSCAN4*, while genes like *CTB-25J19*.1, *TRIM49*, *RFPL1*, *MBD3L2*, *MBD3L3* and *MBD3L5* are coexpressed with *DUX4* in two of the *DUX4*-detected nuclei. Additionally, the nucleus with *DUX4*, *LEUTX* and *ZSCAN4* also expressed *KDM4E*, *TRIM43*, *TRIM43B*, *MBD3L3*, *MBD3L5*, and *RFPL2*. Taken together, the genes expressed in the *DUX4*-detected nuclei may represent early targets of DUX4 which initiate a pathogenic gene regulatory network.

To substantiate the co-expression of *DUX4* and/or DUX4-target genes, we performed RNA FISH on *DUX4* and two representative FSHD-induced genes, *LEUTX* and *SLC34A2*, in day 3 differentiated FSHD2-2 myotubes (Fig 2E). Probes were designed to hybridize to the two regions unique to the *DUX4fl* transcript to ensure the specificity, and we support the specificity with staining for DUX4 protein along with *DUX4* RNA FISH (S11A and S11B Fig). Our *DUX4* probe detected the *DUX4* transcript primarily in the nucleus, possibly reflecting the de novo RNA transcription with some weak signals in the cytoplasm (Fig 2E, S11A and S11B Fig). We observed that ~7% of myotubes have at least 1 *DUX4*-detected nucleus, and that *DUX4*-positive myotubes contain on average 2 *DUX4*-detected nuclei (among on average 15 nuclei per myotube), indicating that even in the permissive patient myotubes, very few nuclei actually express *DUX4*. In these myotubes, however, DUX4 protein spreads to almost all the nuclei (S11B Fig). In contrast to the limited expression of *DUX4* RNA, *LEUTX* and *SLC34A2* RNA transcripts are abundantly present in the cytoplasm in addition to multiple nuclei (Fig 2E). These results are in agreement with snRNA-seq results in which a higher number of nuclei expressing FSHD-induced genes were detected compared to the small number of *DUX4* RNA-positive nuclei (Fig 2A). Taken together, these results suggest that once expressed, DUX4 protein may transcribe target genes in multiple nuclei in the same myotube. Interestingly, we also found that some FSHD myotubes contain *DUX4* transcript but no *LEUTX*, whereas others contain no detectable *DUX4* transcript with abundant signals of *LEUTX* and *SLC34A2* transcripts (Fig 2E and S11C Fig). These results raise the possibility that FSHD-induced gene expression may persist even after *DUX4* transcript is no longer detectable.

### Single-nucleus 3’ end RNA-seq on FSHD2 and control early and late myotubes

**Fig 3:**
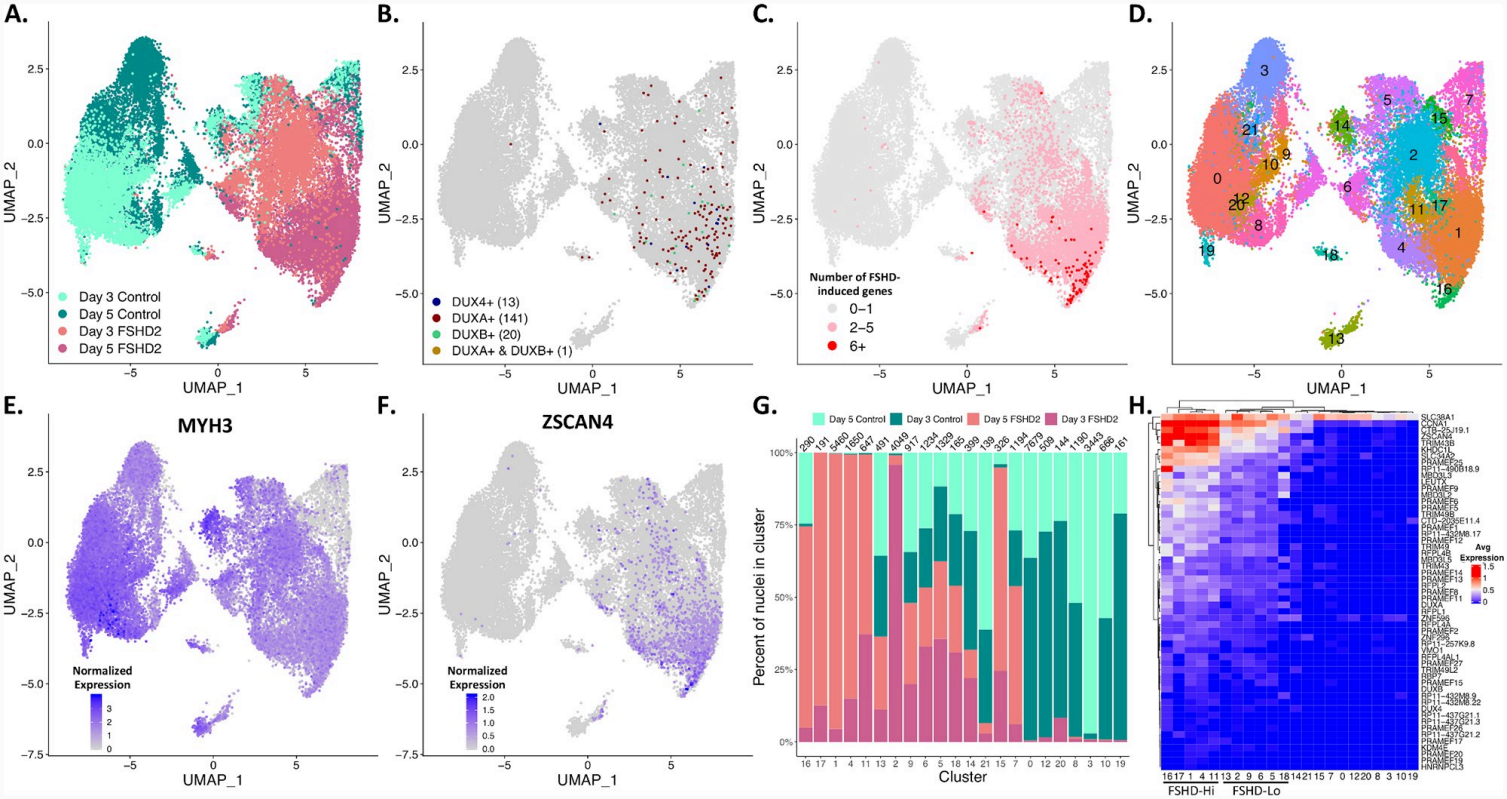
Day 3 and day 5 FSHD2 myotube nuclei cluster based on expression of FSHD-induced genes. **(A)** Day 3 and day 5 myotube nuclei from control-2 and FSHD2-2 plotted on a UMAP based off of expression values from 3’ end sequencing from libraries prepared using the BioRad’s ddSeq. Control and FSHD2 nuclei separate across component 1 (UMAP_1). Day 3 and day 5 nuclei separate within cell type with some mixing. **(B)** UMAP from (A) colored by detection of *DUX4*, *DUXA* and/or *DUXB*. The number of nuclei in which we detect (counts >0) the indicated gene is in parentheses. **(C)** UMAP from (A) colored by the number of FSHD-induced genes detected (counts >0). **(D)** UMAP from (A) colored by cluster determined by shared nearest neighbors (SNN). Each cluster is colored and labeled by its respective number. **(E)** UMAP from (A) colored by expression of the myogenic marker *MYH3*. (F) UMAP from (A) colored by expression of the FSHD-induced gene *ZSCAN4*. **(G)** The percent of control and FSHD2 nuclei from day 3 or day 5 in each cluster from (D). The total number of nuclei in each cluster is indicated above each bar. Colored by cell line and day of differentiation. **(H)** Average expression profiles of the FSHD-induced genes in each cluster. Rows and clusters are ordered and dendrogram is calculated using Euclidean distance.

We identified two distinct populations of FSHD patient nuclei, FSHD-Hi and FSHD-Lo. Since we analyzed a limited number of nuclei using Smart-Seq, we decided to perform additional single-nucleus sequencing in a larger set of nuclei and over two time points in order to address whether the two populations simply reflect different stages of differentiation. We performed 3’ end RNA-seq on two biological replicates of FSHD2-2 and two of Control-2 nuclei from day 3 and day 5 of differentiation using the Illumina SureCell WTA 3’ protocol using the BioRad ddSeq Single Cell Isolator (referred to from now on as “ddSeq”), which allows us isolate thousands of nuclei at a time (Methods). We have 32,273 nuclei which pass our quality filters with an average of 14,139 reads/cell (S12 Fig). We performed the UMAP dimensionality reduction using Seurat on 19,615 genes (Fig 3A). Nuclei separate across the first dimension by disease, and to a lesser extent by differentiation in the second dimension (Fig 3A). To distinguish subpopulations, we cluster the nuclei using shared nearest neighbors (SNN) and find 22 clusters across FSHD2 and control nuclei (Fig 3D). These clusters contain a mix of FSHD2 and control nuclei across differentiation (Fig 3G). We plot the expression of *MYH3* to check that the nuclei are originally from myotubes (Fig 3E). As expected, the majority of nuclei express *MYH3* and were therefore differentiated. However, clusters 15 and 7 have little or no *MYH3* detected and we presume these are either mononuclear cells that did not differentiate given the expression of *MYOD1*, *MYF5 and DES* (S18 Fig) or contaminating non-myogenic cells. We see a similar pattern when looking at expression of other myogenic markers as well (S18 Fig). FSHD2 nuclei seem to have somewhat lower expression of *MYH3* than control across both days of differentiation, which may be biologically significant as was previously noted in that FSHD cells have transcriptome profiles of less differentiated cells [28].

We detect *DUX4* in 13 FSHD2 nuclei, 3 nuclei (0.05%, 3/6152) from day 3 and 10 nuclei (0.1%, 10/9396) from day 5, and they are found spread across multiple clusters (Fig 3B). Higher number of *DUX4*-positive nuclei on day 5 is consistent with the previous studies reporting the increased frequency of *DUX4* expression upon differentiation [16]. Interestingly, the *DUX4*+ nuclei do not cluster with nuclei expressing the highest number of FSHD-induced genes (Fig 3B and 3C). We find a much larger number of nuclei that express *DUXA* and some that express *DUXB*, and these nuclei cluster with nuclei expressing high number of FSHD-induced genes (Fig 3B). Except for one nucleus coexpressing *DUXA* and *DUXB*, the three DUX genes are never coexpressed (Fig 3B).

To identify similar FSHD-Hi and FSHD-Lo populations as found in the full-length RNA-seq data from the Fluidigm C1, we mapped the number of FSHD-induced genes detected per nuclei. Nuclei with 2–5 FSHD-induced genes coexpressed are spread across both day 3 and day 5 FSHD2 myotube nuclei (Fig 3C). Cluster 16 and neighboring clusters have the highest proportion of nuclei with more than 6 FSHD-induced genes deteccted (Fig 3C). *ZSCAN4* expression follows a similar pattern to that of the number of FSHD-induced genes detected, with its highest expression in cluster 16 (Fig 3F). We found *ZSCAN4* to be significantly upregulated starting at day 2 of differentiation in our bulk RNA-seq time-course and therefore its wide spread expression is not surprising. The expression patterns of *ZSCAN4*, particularly in the day 3 FSHD2 nuclei, and the other FSHD-induced genes shows the heterogeneity in the activation of FSHD-induced genes across different nuclei, especially as the day 5 nuclei express *ZSCAN4* more robustly (Fig 3F and S19 Fig). Looking at the average gene expression of all the nuclei for each ddSeq cluster, clusters 16, 17, 1, 4 and 11 have the highest expression of the FSHD-induced genes and are made up primarily of FSHD2 nuclei (Fig 3G and 3H). These ddSeq clusters are akin to the FSHD-Hi cluster from Smart-Seq, and we refer to the FSHD2 nuclei in them collectively as FSHD-Hi (Fig 3H). ddSeq clusters 13, 2, 9, 6, 5 and 18 have moderate expression of the FSHD-induced genes, and cluster separately from the FSHD-Hi clusters (Fig 3H). They also have a large proportion of FSHD2 nuclei and nuclei with 2–5 FSHD-induced genes coexpressed (Fig 3G and 3C). These ddSeq clusters are similar to the Smart-Seq FSHD-Lo group identified from the Fluidigm nuclei, and we therefore label the FSHD2 nuclei in them FSHD-Lo (Fig 3H). Thus, using ddSeq with a larger population of nuclei, we confirmed the presence of two different states of FSHD nuclei “FSHD-Hi and FSHD-Lo”. Importantly, our FSHD-Hi and FSHD-Lo groups includes mixes of both day 3 and day 5 myotube nuclei, suggesting that the differences are not simply attributable to differentiation status (Fig 3G).

### Day 3 FSHD2 myotube nuclei expression patterns are similar across full-length RNA-seq and 3’ end RNA-seq

**Fig 4:**
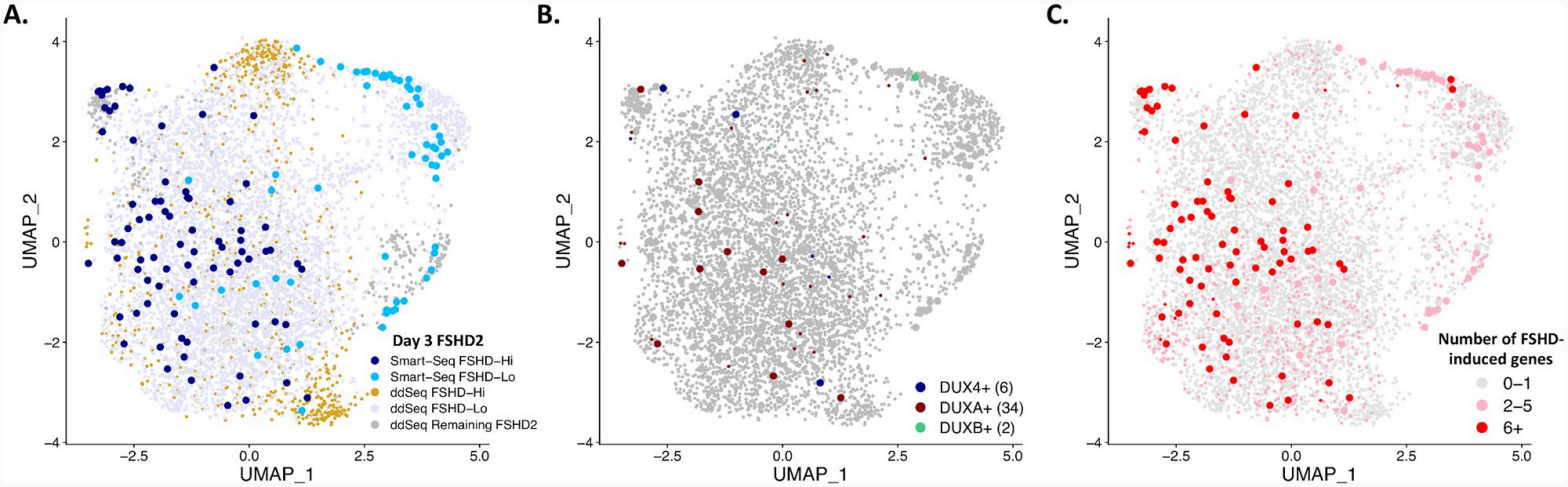
Day 3 FSHD2-2 nuclei from Fluidigm and ddSeq mix. **(A)** UMAP with day 3 FSHD2-2 myotube nuclei from Smart-Seq and ddSeq. Nuclei are colored by technology and classification as FSHD-Hi or FSHD-Lo. **(B)** UMAP from (A) colored by detection (counts >0) of *DUX4*, *DUXA* and/or *DUXB*. The number of nuclei in which we detect the indicated gene is in parentheses. **(C)** UMAP from (A) colored by the number of FSHD-induced genes detected (counts >0).

To make sure that the nuclei from the two sequencing technologies, Smart-Seq and ddSeq, are comparable, we plotted them together on one UMAP (Fig 4A). The nuclei from both technologies overlap, and FSHD-Hi and FSHD-Lo nuclei still separate (Fig 4A). The six *DUX4*+ nuclei from these day 3 FSHD2 samples do not cluster together, nor do they cluster with nuclei with high numbers of FSHD-induced gene detected (Fig 4B, S13 Fig). In this set, no nuclei coexpress *DUX4*, *DUXA* or *DUXB*, perhaps because *DUXB* is expressed later in differentiation as is seen in the bulk timecourse (Fig 1C, S7 Fig). To see if nuclei separate by expression of FSHD-induced genes, we plot the number of FSHD-induced genes and find that nuclei expressing six or more FSHD-induced genes separate to one side of the UMAP, but do not form a distinct cluster (Fig 4C, S13 Fig). Nuclei from the different technologies mix regardless of the number of FSHD-induced genes detected. Given that the nuclei do not separate based on technology, we continue with comparative analysis with the ddSeq data only.

A recent single-cell RNA-seq study also identified a small population of *DUX4* transcript-positive cells in both FSHD1 and FSHD2 patient-derived primary myocytes [29]. In that study, however, myoblast differentiation was induced but myotube fusion was artificially blocked by the use of a calcium chelator [29]. This is in contrast to our study, in which we examined nuclei from unperturbed myotubes using snRNA-seq. Importantly, our approach enables us to uniquely address how *DUX4* expression, even in a single nucleus, results in target gene activation in other nuclei in the same myotube (due to the DUX4 protein spreading) under native condition to distinguish the FSHD-Hi and FSHD-Lo population of cells. We analyzed the expression of 67 DUX4 target genes used in Heuvel, et al. [20, 27] in our FSHD-Hi and FSHD-Lo myotube single nucleus populations. For the Smart-Seq nuclei, all FSHD-Hi nuclei and about 3.3% of FSHD-Lo nuclei highly express at least 5 of these genes (S14 Fig). For the ddSeq nuclei, 5.2% of FSHD-Hi nuclei and 1% of FSHD-Lo nuclei express at least 5 of these genes (S14 Fig). Interestingly, even 1.5% of our ddSeq FSHD2 nuclei excluded from the High and Low populations based on apparent differentiation status express at least 5 of those genes. These percentages are much higher than that in single cell myocyte data (0.2–0.9%) (S14 Fig) [29]. As we confirmed by RNA FISH with DUX4 protein co-staining (Fig 2E and S11 Fig), higher percentages of nuclei expressing more target genes in our study is due to DUX4 protein spreading and target gene activation in multiple nuclei in native myotubes, which is blocked in single nucleus myocytes [29].

We identified that 0.05% of our ddSeq day 3 and 0.1% of our day 5 myotube nuclei express *DUX4*, which is consistent with frequencies observed in other studies (S14A Fig) [16]. In our Smart-Seq data, 2.12% of the day 3 nuclei express *DUX4* at high levels, which is higher than the percentage reported in single cell myocytes (0.2–0.9%) [29] (S14A Fig). Currently unclear is whether blocking myotube fusion interferes with the normal course of myotube differentiation and affects frequency of *DUX4* expression. Taken together, our snRNA-seq analysis captured the extent of target gene expression by the limited expression of *DUX4* in patient myotubes. Our higher-sensitivity Smart-Seq data allowed us to identify the FSHD-Hi and FSHD-Lo populations, and our more robust number of nuclei from the ddSeq data enables us to distinguish the differences between these two populations, possibly representing two different states of patient myotube nuclei.

### FSHD-Hi myotube nuclei turn on cell cycle programs

**Fig 5:**
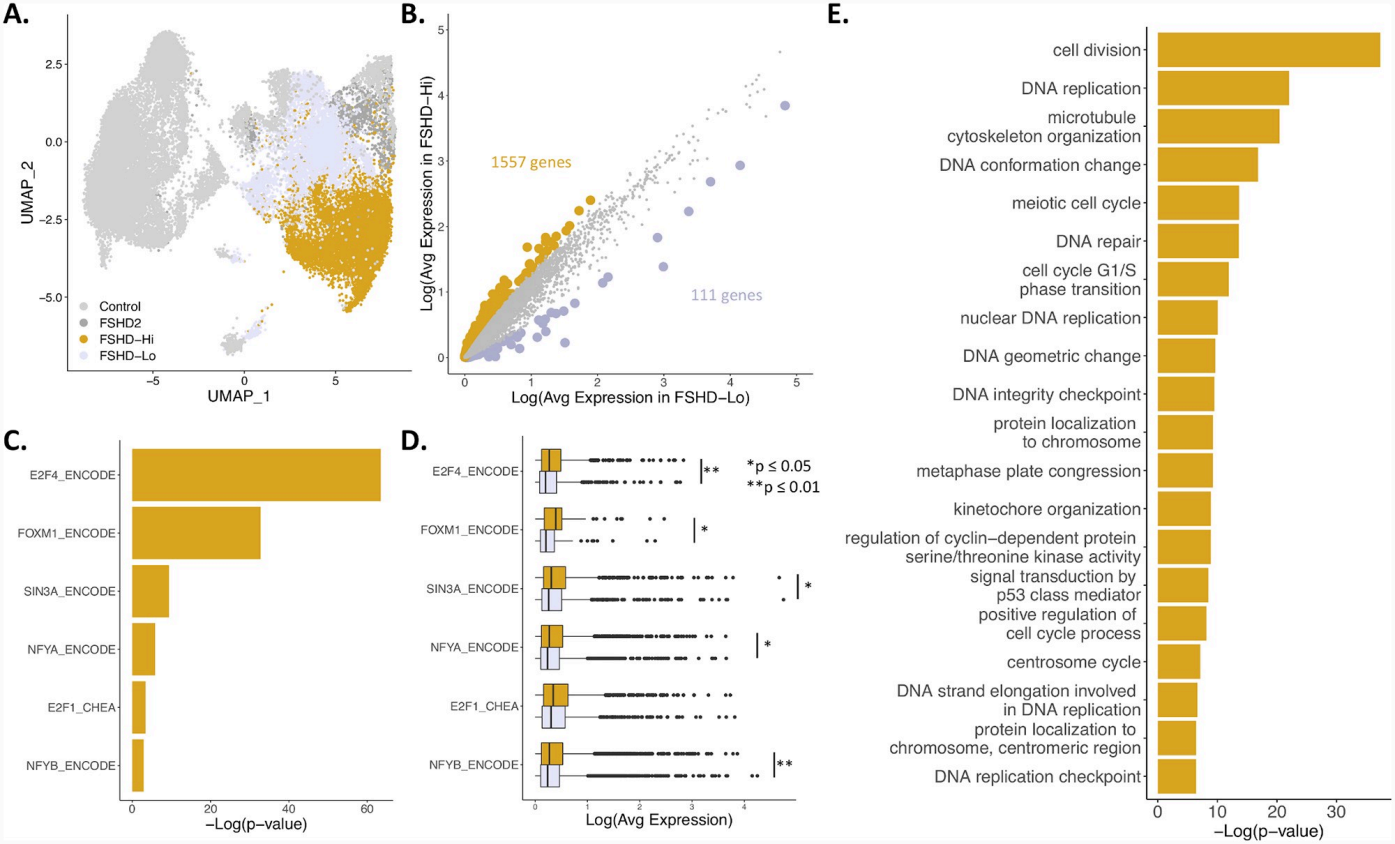
FSHD-Hi nuclei upregulate cell cycle transcription factors and genes. **(A)** UMAP from Fig 3A colored by designation of FSHD-Lo or FSHD-Hi from ddSeq nuclei. **(B)** Scatterplot of average expression of 19,615 genes in the Low and FSHD-Hi populations. Highlighted are genes with FDR <0.05 and abs(log2FC) >1. In gold are genes with higher average expression in FSHD-Hi nuclei. In lavender are genes with higher average expression in FSHD-Lo nuclei. **(C)** Transcription factors and DNA binding proteins with enrichment for binding, as identified from ENCODE and ChEA ChIP-seq datasets, genes significantly higher in the FSHD-Hi population than the FSHD-Lo population. **(D)** Boxplot of average expression of target genes of indicated transcription factors or DNA binding proteins. In gold is the average expression of the targets in the FSHD-Hi nuclei. In lavender is the average expression of the same targets in the FSHD-Lo nuclei. Significance calculated with t-test. All significant differences are marked by asterisks, and p-value is adjusted with FDR. **(E)** Gene ontology terms associated with the 1,557 genes more highly expressed in FSHD-Hi.

To identify genes marking the Low and FSHD-Hi populations, we performed differential expression analysis on 19,615 genes for 6,210 FSHD-Lo nuclei and 8,135 FSHD-Hi nuclei (Fig 5A). We found 1,557 genes significantly more highly expressed in FSHD-Hi and 111 genes more highly expressed in FSHD-Lo (t-test, Benjamini-Hochberg FDR < 0.05) (Fig 5B). Of the 54 FSHD-induced genes, 42 were more highly expressed in FSHD-Hi. SMCHD1 has been shown to regulate the *PCDH* gene clusters, and we find four *PCDH* genes differentially expressed; *PCDH10* and *PCDHGA6* were higher in FSHD-Hi, while *PCDHGB4* and *PCDHGB5* were higher in FSHD-Lo (S4 Table). We also find 149 transcription factors (10% of the FSHD-Hi genes) in FSHD-Hi including 87 zinc fingers and 16 homeobox genes, many of which are important in embryogenesis including several *HOX* genes (S5 Table). We also see 84 cofactors (5% of the FSHD-Hi genes) upregulated including six cyclin genes; *CCNA1*, *CCNA2*, *CCNE1*, *CDK1*, *CDK2*, *CDKN1C* (S5 Table). In contrast, the FSHD-Lo group has 2 transcription factors (2% of the FSHD-Lo genes) and 4 cofactors (4% of the FSHD-Lo genes) upregulated, including *NOTCH3* and *TGFB1* (S5 Table). The mygoenic regulator, *MYOD1*, whose expression decreases during myogeneis, is more highly expressed in the FSHD-Hi group, while *ACTA1*, whose expression increases during myogenesis, is higher in FSHD-Lo. This suggests that although FSHD-Lo has a higher percentage of day 3 FSHD2 myotube nuclei, the FSHD-Hi group has expression of key genes indicative of a less differentiated transcriptomic state.

Additionally, the genes more highly expressed in FSHD-Hi have gene ontology (GO) terms related to cell division and replication (Fig 5E). Included in these categories are many chromatin remodelers and transcription factors involved during the cell cycle. As these myotubes are no longer cycling, these cell cycle related gene products could be altering the genomic landscape in lieu of DNA replication. Additionally, FSHD cells have been shown to have tarnscriptomes of less differentiatined cell states [28]. Activation of these cell cycle genes in the G0 myotubes could be responsible for the less idfferentiatied transcriptomes of FSHD cesll. The GO term “signal transduction by p53 mediator” is also enriched in FSHD-Hi (Fig 5E), and previous studies have shown that DUX4 requires p53 to cause cytotoxicity [30]. These FSHD-Hi nuclei could be activating the p53 pathway and therefore be the disease-driving nuclei in FSHD. GO terms enriched in the FSHD-Lo group include those related to extracelluar structures which has been shown previously to be downregulated in *DUX4* expressing cells (S15 Fig) [22].

To identify regulators key to the genes upregulated in the FSHD-Hi population, we looked for enrichment of transcription factors and other DNA binding proteins that bind these genes based off of ChIP-seq data from two genomic databases, ENCODE and ChEA (Fig 5C). Five transcription factors, E2F1, E2F4, FOXM1, NFYA and NFYB, and one corepressor, SIN3A, are statistically enriched for regulating the FSHD-Hi genes. All of these are involved in cell cycle gene regulation, which is consistent with the GO terms identified for these genes. *FOXM1* and *E2F1* are both upregulated in FSHD-Hi nuclei as well (Fig 5B, S4 Table). The target genes for five of these transcriptional regulators, all but E2F1, show a significant difference in expression between FSHD-Hi and FSHD-Lo (Fig 5D). E2F4 represses genes which are upregulated by E2F1 during the G1 to S phase transition, which may explain why we see E2F4 target genes as significantly different between the two groups but not E2F1 [31]. Additionally, we do not detect this upregulation of cell cycle genes other than *CCNA1* in the bulk RNA-seq time-course (Fig 1B and S5 Fig), which emphasizes that this upregulation is specific to these FSHD-Hi nuclei.

### DUXA regulates FSHD-induced genes

Given that *DUX4* expressing nuclei did not cluster with the nuclei expresing the highest amount of FSHD-induced genes (Fig 3B and 3C), we searched for other widespread transcriptional regulators that could be regulating the FSHD-induced genes in a wider set of nuclei. A DUX4 target gene, *DUXA*, is highly upregulated in FSHD2 and detected in a large number of nuclei and we therefore looked for binding sites of these two transcription factors, DUX4 and DUXA, in the promoter regions (-1.5 kb to +0.5 kb) of *DUX4*, *DUXA*, *ZSCAN4* and *LEUTX* to see if they could be regulating themselves and other FSHD-induced genes. A ChIP-seq binding motif is available for DUX4, and an HT-SELEX motif is available for DUXA (S16A and S16B Fig) [32]. Not surprisinlgy, DUX4 has one binding site in each of the FSHD-induced genes we looked at, two for *LEUTX*, and one for itself. DUXA has one binding site for itself, one in the promoter of *LEUTX*, and two for *ZSCAN4*. The DUX4 and DUXA binding sites overlap in the *DUXA* promoter, and for one of the sites for *LEUTX* and one for *ZSCAN4* (S16C Fig). Since the binding sites overlap, DUXA, once expressed, may regulate these DUX4 target genes after DUX4 is no longer present.

**Fig 6:**
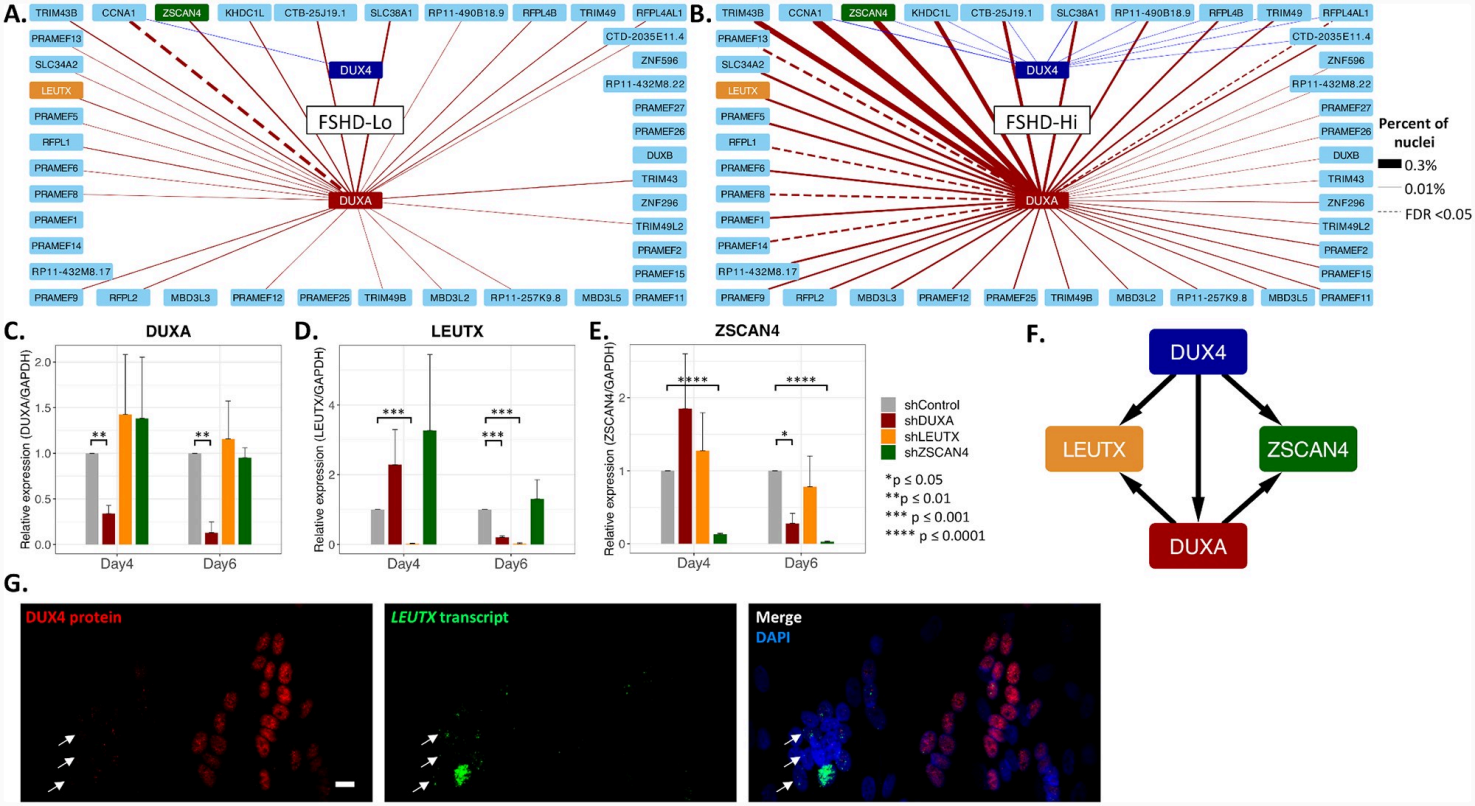
DUXA regulates FSHD-induced genes. Coexpression network of 41 FSHD-induced genes which are coexpressed with *DUX4* and/or *DUXA* in **(A)** the ddSeq FSHD-Lo population and **(B)** the ddSeq FSHD-Hi population. Line thickness is the percent of nuclei coexpressing the two genes. Red lines represent coexpression with *DUXA*. Blue lines represent coexpression with *DUX4*. Dashed lines indicates FDR <0.05 using Fisher’s exact test. **(C-E)** RT-qPCR analyses of the effects of lentiviral shRNA depletion of three DUX4 target transcription factors. Relative RNA expression of **(C)** *DUXA*, **(D)** *LEUTX*, and **(E)** *ZSCAN4* on days 4 and 6 of differentiation in FSHD2-2 cells following depletion of each gene product as indicated is shown. Expression measured by qPCR, and values are normalized to *GAPDH* expression and the non-targeting shControl. Significance calculated with t-test, and n = 3 for each condition. All significant differences are marked by asterisks. Color indicates the shRNA used as listed on the right. **(F)** Proposed model for DUXA regulating FSHD-induced genes in addition to DUX4. **(G)** Expression of DUX4 protein and its downstream target gene are not always concordant. Immunofluorescence detection of DUX4 protein (red) and RNAScope for *LEUTX* transcript (green) in FSHD2-2 day 7 myotubes. Examples of a *LEUTX transcript*-positive myotube with no significant DUX4 protein (left) and a DUX4 protein-positive myotube with very little LEUTX transcript signal (right) are shown. Bar, 10 μm. DAPI is in blue. Nuclei with *LEUTX* transcripts with no DUX4 protein are indicated by white arrows.

To further analyze the relationship between *DUX4*, *DUXA* and other FSHD-induced genes, we look at the coexpression of DUX4 and DUXA with the FSHD-induced genes in the FSHD-Hi and FSHD-Lo populations (Fig 6A and 6B). In the FSHD-Lo, we see *DUX4* coexpressed with *CCNA1* only (Fig 6A). However, *DUXA* is coexpressed with a 26 FSHD-induced genes in the low population. In the FSHD-Hi population, we see *DUX4* coexpressed with 10 FSHD-induced genes, while *DUXA* is coexpressed with 41 FSHD-induced genes (Fig 6B). Assuming that the nuclei in which we detect *DUX4* are the first to express *DUX4* in their respective myotubes, these ten genes coexpressed with *DUX4* may be its early targets. However, we cannot rule out that these differences could be attributable to the detection sensitivity of the technology and to the difference between the number of *DUX4* and *DUXA* expressing nuclei detected. *ZSCAN4* is coexpressed with *DUX4* and to a larger extent with *DUXA* (Fig 6B). *LEUTX* appears to be coexpresssed primarily with *DUXA* in both the FSHD-Hi and FSHD-Lo populations (Fig 6A and 6B). As described earlier, we observed that some myotubes express *LEUTX* with apparent lack of *DUX4* transcript (Fig 2E and S11C Fig). However, this may be due to persistent DUX4 protein. Thus, we performed DUX4 protein immunostaining combined with *LEUTX* RNA FISH (Fig 6G and S11B Fig). While DUX4 protein can be detected in multiple nuclei within the same myotube expressing *LEUTX* (S11B Fig), we also found that in some myotubes the levels of DUX4 protein and *LEUTX* transcript expression are discordant (Fig 6G). Indeed, in some nuclei with *LEUTX* expression, no significant DUX4 protein was detected, raising the possibility that *LEUTX* may be transcribed in the absence of DUX4 protein (Fig 6G).

To further assess the relationship between DUX4 target transcription factor genes, *DUXA*, *LEUTX* and *ZSCAN4*, we transfected FSHD2-2 myoblasts with shRNAs against *DUXA*, *LEUTX* and *ZSCAN4* and measured their gene expression after 4 and 6 days of differentiation (S17 Fig). Interestingly, RT-qPCR results reveal that while depletion of *DUXA* has no significant effect on *LEUTX* and *ZSCAN4* expression on day 4, it significantly suppressed their expression on day 6 (Fig 6C, 6D and 6E). Depletion of *LEUTX* or *ZSCAN4* did not have any significant effect on *DUXA* expression on either day 4 or day 6 (Fig 6C). The results demonstrate that in addition to DUX4, DUXA can regulate the expression of *LEUTX* and *ZSCAN4* (Fig 6F). Differential effects on days 4 and 6 strongly suggest that these genes are initially activated by DUX4, but once sufficient amount of DUXA is induced, their expression is further promoted by DUXA. Thus, DUXA may function to amplify and sustain the DUX4 signal in this way, providing a self-supporting network of gene disregulation that can lead to pathogenesis regardelss of the temporoary expression of *DUX4* consistent with the long-standing observation in previous studies that FSHD-induced gene expression is easier to detect in patient muscle cells than the *DUX4* transcript itself.

## Discussion

Using our time-course bulk RNA-seq analysis of control and FSHD2 patient myoblast differentiation, we defined a set of 54 genes that are specifically induced in FSHD2 as “FSHD-induced genes”. Those genes largely overlap with previously defined downstream targets of DUX4 [22, 25] though we cannot rule out the possible contribution of the *SMCHD1* mutation. Single-cell and single-nucleus RNA-seq on two different platforms revealed that FSHD2 myotube nuclei express higher FSHD-induced genes than myoblasts or control myotube nuclei. Importantly, we were able to identify *DUX4* transcript-positive nuclei, which were not detected in our bulk RNA-seq. We further identified two populations of FSHD2 myotube nuclei, FSHD-Hi and FSHD-Lo, based on the expression of FSHD-induced genes. We found that FSHD-Hi nuclei also upregulate cell cycle genes and identified a set of transcriptional regulators that may contribute to this upregulation. We found evidence that DUXA affects expression of the DUX4 target genes *LEUTX* and *ZSCAN4* in later days, which raises the possibility that DUXA may be important for DUX4 signal amplification by contributing to the regulation of DUX4 target genes.

While FSHD-Hi nuclei express markers of differentiated myotubes and have a higher proportion of day 5 nuclei than FSHD-Lo, they exhibit higher expression of *MYOD1* and lower expression of *ACTA1* than FSHD-Lo. Thus, FSHD-Hi nuclei appear to have transcriptomic markers of a less differentiated state, which may be consistent with a previous observation in a mouse model of FSHD [28]. Accordingly, we found that cell cycle genes are specifically upregulated in FSHD-Hi nuclei, and five transcription factors, E2F1, E2F4, FOXM1, NFYA and NFYB, and one corepressor, SIN3A, are statistically enriched for regulating these genes. Interestingly, some of these factors have been previously linked to FSHD-related gene regulation. SIN3A complexes with HDACs and TET proteins and appears to be involved in *DUX4* repression [33, 34]. NF-Y, made up in part by NFYA and NFYB, binds to HERV LTR repeats which are activated in FSHD [3, 35]. E2F4, E2F1 and FOXM1 are all part of the DREAM complex which regulates cell cycle genes [31]. E2F1 activates a DUX4-target gene *CCNA1*, and both E2F1 and FOXM1 are regulated by phosphorylation by CDK2 complexed with cyclin A [31, 36], which are both upregulated in FSHD-Hi nuclei. Thus, these cell cycle transcriptional regulators may contribute to FSHD-associated gene dysregulation. How these cell cycle-related genes in a subset of post-mitotic, multinucleated myotubes contribute to pathogenesis in FSHD is currently unclear.

Small populations of DUX4-positive myotubes are thought to drive pathogenesis in FSHD [2–4]. We found 0.1% (16/15,687) of nuclei expressed *DUX4* using single-nucleus RNA-seq which is lower than the reported 0.5% (1/200 in myotube nuclei) [8, 10, 16, 27] possibly due to variability in expression levels of *DUX4* between individuals. However, the percentage of DUX4-affected nuclei we found (3.7%, 583/15,687) is higher than that reported in FSHD single myocytes (0.55%, 27/4902) [29]. Our high-resolution single-cell and single-nucleus dataset is the first to observe the endogenous expression of *DUX4* in a small number of FSHD2 myotube nuclei with wider expression of target genes. Our snRNA-seq and immuno-RNA FISH results demonstrate that one or two nuclei expressing *DUX4* transcripts appears to produce sufficient DUX4 protein to spread to multiple nuclei, consistent with a previous study [16, 22], and possibly initiate target gene expression.

Previous studies suggested a feedback loop between DUX4 and its target genes to further increase DUX4 expression via (1) DUX4-mediated proteolytic degradation of UPF1 and inhibition of nonsense-mediated RNA decay resulting in stabilization of DUX4 mRNA [37], and (2) the DUX4 target MBD3Ls binding to D4Z4 and relieving DUX4 repression [34]. Additionally, alternate histones which are targets of DUX4 have been shown to continue the expression of DUX4 target genes [27]. In the current study, we provide support for DUXA as another regulator of DUX4 target genes which may amplify the DUX4 gene network. Although DUXA is a DUX4 paralog [25] and has been identified as a DUX4 target gene in human patient muscle cells [20], no study reports its specific functions in FSHD. DUXA is a transcription factor with two homeobox domains like DUX4, and it binds to a 10 bp motif similar to DUX4 [24, 29]. Importantly, our results indicate that *DUXA* upregulates *LEUTX* and *ZSCAN4* in late but not early in differentiation, suggesting a possible two-step mechanism for upregulation of these DUX4-target transcription factors; first by DUX4 then DUXA. In support of this, we found that *LEUTX* is present in nuclei with no significant DUX4 protein present. Given that *DUXA* is much more widely expressed together with FSHD-induced genes in our analysis, we propose that DUXA may drive an FSHD-specific pathogenic program by binding and activating a pool of DUX4 targets, therefore reinforcing the DUX4-induced gene network in patient myotube nuclei.

Previous studies indicated that DUX4 expression leads to p53-dependent apoptosis within 20 hours of initial expression [15, 22, 30]. We observed, however, continuous upregulation of DUX4 target genes over 6 days without any overt cytotoxic phenotype or apoptotic transcriptomic signature. This suggests that DUX4 upregulation may not be immediately toxic in the endogenous context. We hypothesize that sporadic endogenous *DUX4* expression may be relatively short-lived, and that downstream DUX4 target genes, such as DUXA, may amplify and/or reinforce the FSHD-induced gene network in addition to or in place of DUX4 eventually leading to myotoxicity and dystrophy. If this is the case, it is possible that therapeutics targeting DUX4 or *DUX4* expression may limit initiation of FSHD in new tissue but may not stop muscle wasting in already disease-activated tissue, and targeting transcription factors downstream of DUX4 may be necessary.

Our time-course bulk RNA-seq and single-cell/-nucleus RNA-seq of primary control and FSHD2 myoblasts and myotubes addressed FSHD-specific gene expression during differentiation. Single-nucleus RNA-seq demonstrated the heterogeniety and disease-specific transcriptomic changes of patient myotube nuclei with high or low expression of *DUX4* and FSHD-induced genes.Our results provide strong evidence that *DUX4* transcript expression in one or two nuclei can result in a high expression of downstream target genes in the entire myotube, which may be mediated by DUX4 protein spreading to multiple nuclei as well as signal amplification by downstream target transcription factors, such as DUXA. Although our current study is limited to FSHD2 primary myocytes from tibialis anterior, our strategy should be effective in further analyzing FSHD pathophysiology during different stages of muscle differentiation and in biopsies and muscle types with different sensitivities to the disease at a single nucleus resolution.

## Materials & methods

### Human myoblast culture and differentiation

Human control and FSHD2 myoblast cells from patient quadricep and tibia biopsies were grown on dishes coated with collagen in high glucose DMEM (Gibco) supplemented with 20% FBS (Omega Scientific, Inc.), 1% Pen-Strep (Gibco), and 2% Ultrasor G (Crescent Chemical Co.) [21]. Upon reaching 80% confluence, differentiation was induced by using high glucose DMEM medium supplemented with 2% FBS and ITS supplement (insulin 0.1%, 0.000067% sodium selenite, 0.055% transferrin; Invitrogen). Fresh differentiation medium was changed every 24hrs.

### Bulk, single-nucleus and single-cell RNA-seq library preparation and sequencing

For bulk RNA-seq for the time-course, total RNA was extracted by using the RNeasy kit (QIAGEN). Between 19 and 38 ng of RNA were converted to cDNA using the Smart-Seq 2 protocol [38]. Libraries were constructed with the Nextera DNA Library Prep Kit (Illumina) for control-3, control-4, and FSHD2-2 libraries, and the Nextera DNA Flex Library Prep Kit (Illumina) for control-1, control-2 and FSHD2-1 libraries. Libraries were sequenced on the Illumina NextSeq500 platform using paired-end 43 bp mode with 15 million reads per sample.

Full-length single-cell and single-nucleus RNA-seq was performed according to [21] using the Fluidigm C1 with the following modifications. Myotube single nuclei were isolated from mononucleated cells (MNCs) by washing a 6 cm dish once with trypsin, then adding trypsin for about 5 min until myotubes lifted off the plate and MNCs were still attached. Cells were initially pelleted at 2000 rpm for 2 min and resuspended in lysis buffer with 0.02% IGEPAL CA-630. Lysis was done for 3 minutes, filtered and spun at 4000 rpm for 1 minute. Nuclei were captured on medium IFCs (10–17 um) at a density between 340 and 640 nuclei/ul in a volume of 10 ul. Visual confirmation was aided with the LIVE/DEAD kit (Thermo Fisher Scientific), and cDNA was normalized to approximately 0.1 ng/ul for tagmentation and library prep. Libraries were quality-controlled prior to sequencing based on Agilent 2100 Bioanalyzer profiles and normalized using the KAPA Library Quantification Kit (Illumina). Libraries were sequenced on the Illumina NextSeq500 platform using paired-end 75 bp mode with 1–3 million reads per sample for full-length RNA-seq single-cell and single-nucleus libraries.

Single-nucleus 3’ end RNA-seq libraries from nuclei isolated on the ddSeq Single Cell Isolator (BioRad) were prepared as follows. Myotubes from day 3 or day 5 of differentiation were isolated from mononucleated cells (MNCs) by washing a 35 mm dish once with trypsin, then adding trypsin for about 5 min until myotubes lifted off the plate and MNCs were still attached. Cells were washed once in 1X PBS + 0.1% BSA, and the nuclei were prepared according to [39] with the following modifications. We used 0.2 U/ul RNasin Plus RNase Inhibitor (Promega) for the cell lysis buffer and nuclei storage buffer, and nuclei were filtered through a 40 um filter (Falcon) after isolation. Nuclear isolation and quality were assessed by staining with ethidium homodimer. Nuclei were loaded onto the ddSeq Single Cell Isolator (BioRad) for droplet generation, and libraries were prepared using the SureCell WTA 3’ Library Prep Kit (Illumina). Libraries were sequenced on the Illumina NextSeq500 platform using PE 68 bp for read 1 and 75 bp for read 2 with a custom primer with around 370 million reads for four samples.

### RNA FISH (Fluorescent *in situ* hybridization targeting ribonucleic acid molecules) by RNAScope

FSHD2-2 myoblasts were seeded in micro-slide eight-well plates at ~8x10^4^ cells per well, and differentiation was initiated ~20hrs later. After 3 or 7 days, as indicated, of differentiation, cells were fixed with 10% neutral buffered formalin at room temperature for 30 min, and the RNA FISH experiments were performed using the RNAScope fluorescent Multiplex system (Advanced Cell Diagnostic Inc.) according to the manufacturer’s instructions. For costaining of immunofluorescent (IF) staining and RNAScope, cells were permeabilized with 0.5% Triton X-100 for 5 min at 4°C between fixation and dehydration process, then DUX4 (Abcam, ab124699) IF was performed as previously described [40]. Probe-Hs-DUX4-C1, Probe-Hs-LEUTX-C2, were custom-designed to avoid crossreactivity to related homologs (for *DUX4* probe set, see S11A Fig). Probe-Hs-SLC34A2-C3 was also used. Images were acquired using a Zeiss LSM 510 META confocal microscope. A technical consideration should be made that due to the process of IF and RNAScope costaining that much of the cytoplasmic RNAScope signal is washed out.

### Quantification of differentiation index in myosin heavy chain 1 (MYH1) stained control and FSHD2 cells

Control-2 and FSHD2-2 cells were fixed with 4.0% paraformaldehyde in PBS for 10 min at room temperature, and cells were permeabilized with 0.5% Triton X-100 for 5 min at 4°C. Then MYH1 (ABclonal, Inc., A6935) IF was performed as previously described [40]. Differentiation index is defined as the number of nuclei in myotubes expressing MYH1 divided by the total number of nuclei in a field. We determined the differentiation index by counting at least 600 nuclei from 3 random fields on the coverslip which was fixed at each time point of differentiation.

### shRNA depletion of DUX4 target genes

Lentiviruses carrying shRNA plasmids for each DUX4 target gene: *DUXA* (5’-CTAGATTACTTCTCCAGAGAA-3’, TRCN0000017664), *LEUTX* (5’-CCTGGAATCTCTGATGCAAAT-3’, TRCN0000336862), *ZSCAN4* (5’-CCCAAGATACTTCCTTAGAAA-3’, TRCN0000016848) and an shRNA non-targeting control (Sigma-Aldrich, SHC002) were made in 293T cells using Lipofectamine 3000. The cells were transfected with 2 ug of shRNA plasmids, 1.5 ug of pCMV plasmids, and 0.5 ug of pMP2G plasmids. The media was changed after 24 hours. The lentiviruses were harvested at 48 hours and 72 hours post-transfection. FSHD2-2 myoblasts were infected once at 32 hours and once at 8 hours prior to addition of differentiation media. The myoblasts were selected for plasmid integration using puromycin. RNA was extracted using RNeasy kit (Qiagen) at days 4 and 6 of differentiation. Approximately 16 ng of RNA was converted to cDNA using SuperScript VILO (Invitrogen), and expression quantitation of *DUXA*, *LEUTX*, *ZSCAN4*, and *GAPDH* was done via RT-qPCR using SYBR green (Invitrogen) and the primers listed in Table 1.

**Table 1:** Primer sequences used for qPCR.

Primers	Sequence
ZSCAN4 Fwd	5’–TGGAAATCAAGTGGCAAAAA– 3’
ZSCAN4 Rev	5’–CTGCATGTGGACGTGGAC– 3’
LEUTX Fwd	5’–GGGAAACTGGCTTCAAAGCTA– 3’
LEUTX Rev	5’–TGATGGCCGTGTCTGCATTT– 3’
DUXA Fwd	5’–GCCTTACCCAGGTTATGCTACC– 3’
DUXA Rev	5’–TGGAATCCGTGCCTAGCTCTT– 3’
GAPDH Fwd	5’–TCGACAGTCAGCCGCATCT– 3’
GAPDH Rev	5’–CTAGCCTCCCGGGTTTCTCT– 3’

### RNA-seq data processing

Raw reads from both bulk RNA-seq and single-cell and single-nucleus RNA-seq were mapped to hg38 by STAR (version 2.5.1b) [41] using defaults except with a maximum of 10 mismatches per pair, a ratio of mismatches to read length of 0.07, and a maximum of 10 multiple alignments. Quantitation was performed using RSEM (version 1.2.31) [42] by defaults with gene annotations from GENCODE v28, and results were output in transcripts per million (TPM). Myoblast cells were kept for downstream analysis if *desmin* expression was > = 1 TPM, *MYOG* <1 TPM, number of expressed genes was more than 500 and expression level of *GAPDH* was higher than 100 TPM. Myotube nuclei were kept for downstream analysis if *MYOG* expression was > = 1 TPM, number of expressed genes was more than 500 and expression level of *GAPDH* was higher than 100 TPM. We only kept cells and nuclei with a uniquely mapped efficiency higher than 45%. For differential gene expression analysis in differentiation time-course, protein coding and long non-coding RNA genes with greater than 5 TPM in both replicates in at least one timepoint and with greater than 1 TPM for both reps for both cell lines of the same disease and day were kept. Genes were TMM normalized using edgeR (version 3.18.1) [43] and log2-transformed. For the bulk RNA-seq time-course, Batch correction was performed using ComBat from sva (version 3.32.1) and scaled for two batches which used different library prep kits; control-3, control-4, FSHD2-2 for one batch, and control-1, control-2, FSHD2-1 for the second. LogFC and p-values of FSHD-induced genes was calculated using edgeR with p-value <0.05. Clustering of genes across the time-course was done by using maSigPro using an r-squared of 0.66 [23]. Comparisons in Figs 5D, 6C, 6D and 6E, S9 Fig were done using a t-test, and FDR was used where indicated (stats package version 3.6.1).

Sequencing data from 3’ end RNA-seq was demultiplexed using ddSeekR [44]. Nuclei with at least 500 UMIs detected were mapped using STAR (version 2.5.1b) [41] and quantitated using RSEM (version 1.2.31) [42] with the *rsem-calculate-expression* with options—*star* and—*estimate-rspd*. We kept nuclei with ≥150 genes detected and <20% mitochondrial reads. Genes detected in at least 5 nuclei were kept for downstream analysis. The data was loaded into Seurat (version 3.1.0) and normalized using the SCTransform function [42, 43]. Seurat was also used to create UMAPs, determine clusters and calculate average expression. Heatmap of average expression was created using ComplexHeatmap (version 2.0.0) [45]. For overlap of full-length RNA-seq data with 3’ end RNA-seq data, we apply SCTransform to both sets individually, then use the integration pipeline in Seurat to combine the datasets [46,47]. Differentially expressed genes were called using a t-test and FDR calculated from the stats (version 3.6.1) package with an FDR cutoff of 0.05 and a log2FC cutoff of 1. Fold change between the groups was calculated using average expression calculated in Seurat. Gene ontology analysis was done by using Metascape [48] with the whole genome as the background set and an FDR <0.05. Transcription factor and DNA binding protein enrichment was done using enrichR (version 2.1) [49] with an adjusted p-value cutoff of 0.05. Transcription factors and cofactors identified from AnimalTFDB (version 3.0) [50]. Gene coexpression networks were plotted by using Cytoscape [51] using counts or TPM >0.

### Binding site analysis of DUXA and DUX4

We used binding motifs from HOCOMOCO v11 [32] for DUX4 and DUXA as input into HOMER (version 4.10) using the scanMotifGenomeWide.pl command for hg38 [52]. Motif logos were generated using LogOddsLogo [53].

### Reanalysis and comparisons of previously published data

Fastq files from [20, 22, 25] (Table 2) were obtained from SRA and mapped and quantitated as described above. We kept genes with greater than 1 TPM either for all experimental or FSHD samples or control samples. Genes with a logFC >2 and p-value <0.01 as calculated by edgeR were considered differentially expressed. For comparisons with [29], we report the 95% confidence interval calculated using prop.test from stats (version 3.6.1). We use the DUX4-affected cell counts found in Supplemental table 4 of [29].

**Table 2:** Accession numbers for published datasets used in this paper.

Reference	Sample Name	SRA
[24]	Sample_1-MB135_HDUX4CA_nodox_rep1	SRR4019004
[24]	Sample_2-MB135_HDUX4CA_WITHdox_rep1	SRR4019005
[24]	Sample_3-MB135_HDUX4CA_nodox_rep2	SRR4019006
[24]	Sample_4-MB135_HDUX4CA_WITHdox_rep2	SRR4019007
[24]	Sample_5-MB135_HDUX4CA_nodox_rep3	SRR4019008
[24]	Sample_6-MB135_HDUX4CA_WITHdox_rep3	SRR4019009
[22]	FSHD_1_1_neg	SRR2020583
[22]	FSHD_1_2_neg	SRR2020584
[22]	FSHD_2_2_BFP	SRR2020585
[22]	FSHD_2_3_BFP	SRR2020586
[22]	FSHD_1_3_neg	SRR2020587
[22]	FSHD_1_1_BFP	SRR2020588
[22]	FSHD_1_2_BFP	SRR2020589
[22]	FSHD_1_3_BFP	SRR2020590
[22]	FSHD_2_1_neg	SRR2020591
[22]	FSHD_2_2_neg	SRR2020592
[22]	FSHD_2_3_neg	SRR2020593
[22]	FSHD_2_1_BFP	SRR2020594
[20]	Control_20_Mt	SRR1398556
[20]	Control_21_Mb	SRR1398557
[20]	Control_21_Mt	SRR1398558
[20]	Control_22_Mb	SRR1398559
[20]	Control_22_Mt	SRR1398560
[20]	FSHD2_12_Mt	SRR1398561
[20]	FSHD2_14_Mb	SRR1398562
[20]	FSHD2_14_Mt	SRR1398563
[20]	FSHD2_20_Mb	SRR1398564
[20]	FSHD2_20_Mt	SRR1398565
[20]	FSHD1_4_Mb	SRR1398566
[20]	FSHD1_4_Mt	SRR1398567
[20]	FSHD1_6_Mb	SRR1398568
[20]	FSHD1_6_Mt	SRR1398569

## Supporting information

S1 Fig Quality metrics of RNA-seq time-course data. Control and FSHD2 time-course quality metrics for **(A)** the number of uniquely mapped reads, **(B)** mapping efficiency, **(C)** the number of genes detected (TPM> = 1).
(TIF)

S2 Fig Expression of *DUX4-fl* in FSHD2-2 cells. **(A)** Nested RT-PCR analysis of *DUX4-fl* expression in differentiated FSHD2-2 cells at day 3. The PCR product was sequenced to confirm its identity. The nested PCR was done using the primer sets (182–183 and 1A–184) previously published [2]. **(B)** FSHD2-2 cells were incubated in differentiation medium for the indicated days, and RT-qPCR was used to assess DUX4 mRNA expression during differentiation. Left, RT-qPCR data are normalized to GAPDH and the graph shows the relative abundance of *DUX4* mRNA at indicated time points. Error bars are standard deviation. P values comparing to Day 1 were shown. At Day 1, the *DUX4* mRNA is so low that nonspecific PCR product was amplified. Other PCR product was verified by sequencing. The qPCR primers are 5'-CCCAGGTACCAGCAGACC-3' and 5'-TCCAGGAGATGTAACTCTAATCCA-3’ [9]. Right: the qPCR products were run on the gel and their identity was confirmed by sequencing (data not show).
(TIF)

S3 Fig Principal component analysis (PCA) on control and FSHD2 myoblast differentiation time-course. **(A)** PCA with PC1, PC2 and PC3 for FSHD2 and control myoblasts from tibialis anterior. PC2 further explains the expression variance across differentiation. **(B)** PCA with PC1, PC2, and PC3 for controls from tibialis anterior (TA) and controls from quadricep (quad). PC2 and PC3 combined explain the expression variance for muscle source and sex. Gene expression level was measured each day for duplicates by using RNA-seq. Cell types are labeled by shape, and time-points are labeled by color.
(TIF)

S4 Fig Principal component analysis (PCA) on control and FSHD2 myoblast differentiation time-course. **(A)** PCA with PC1, PC2, and PC3 for FSHD2, controls from tibialis anterior (TA) and controls from quadricep (quad). PC2 further explains the expression variance across differentiation. **(B)** PCA with PC1, PC3, and PC4 for FSHD2, controls from tibialis anterior (TA) and controls from quadricep (quad). PC3 and PC4 account for variation in gene expression between FSHD2 and control samples. Gene expression level was measured each day for duplicates by using RNA-seq. Cell types are labeled by shape, and time-points are labeled by color.
(TIF)

S5 Fig Genes variable across time but not between FSHD and control form two clusters. **(A)** Cluster 1 gene decrease during differentiation. **(B)** Cluster 2 gene increase during differentiation. **(C)** Quantification of differentiation index in myosin heavy chain1(MYH1) stained control-2 and FSHD2-2 myoblast cell lines for days 0, 3 and 5 of differentiation. Differentiation index is defined as the number of nuclei in myotubes expressing MYH1 divided by the total number of nuclei in a field. We determined the differentiation index by counting at least 600 nuclei from 3 random fields on each coverslip which was fixed at indicated days after differentiation. Myotubes with any detectable MYH1 signal are considered positive, and the signal strength of MYH1 staining is not taken into consideration. Statistically significant delay of differentiation was observed in FSHD myocytes compared to the control used on day 3 (~70% as opposed to 90%). On day 5, differentiation index is still lower in FSHD than control but the difference is no longer statistically significant. **(D)** Representative images of differentiation marker MYH1 (red) staining of days 0, 3 and 5 of differentiation in control-2 and FSHD2-2 cells. Bar, 10 μm. DAPI is in blue.
(TIF)

S6 Fig Venn diagram of FSHD-induced genes from this study and published FSHD and DUX4 induced genes. **(A)** Overlap of 53 of the 54 genes upregulated during FSHD2 differentiation time-course from myoblasts to myotubes compared to 625 genes upregulated in myoblasts with doxycycline induced *DUX4* expression [25] and to 587 genes upregulated in *DUX4* expressing myotubes over non-expressing myotubes [22]. Published data was reanalyzed using the same analysis pipeline (Methods). **(B)** Overlap of 54 genes upregulated during FSHD2 differentiation time-course from myoblasts to myotubes compared to 91 genes upregulated in FSHD primary myoblasts and myotubes compared to control [20]. Published data was reanalyzed using the same analysis pipeline (Methods).
(TIF)

S7 Fig Fold change heatmap of FSHD-induced genes for FSHD2-1 and FSHD2-2 vs control-1 and control-2. All logFC with p <0.05 are shown for comparisons of FSHD2 to control for each day of differentiation.
(TIF)

S8 Fig Overview of single-cell and single-nucleus samples from Fluidigm and comparison with time-course. **(A)** Summary of single cells and single nuclei collected for sequencing. Single cells from myoblasts were selected to be *desmin*(+) *MYOG*(-) cells and retained for downstream analysis. Single nuclei from myotubes were selected to be *desmin*(+) *MYOG*(+) nuclei and retained for downstream analysis. Average number of reads, average number of mapped reads, and median number of genes detected are given per cell or nucleus for each sample. **(B)** Principal component analysis (PCA) of Control-1 and FSHD2-2 myoblast differentiation time-course. Gene expression level was measured each day for duplicates by using RNA-seq. Cell types are labeled by shape, and time-points are labeled by color. **(C)** Incremental PCA on pooled Control-1 single cells and pooled FSHD2-2 single nuclei as well as bulk Control-1 and FSHD2-2 differentiation time-courses with the same dimensions as the PCA in (B).
(TIF)

S9 Fig Differences in the number of FSHD-induced genes from the time-course which are detected across sample types. Comparison of the number of FSHD-induced genes detected (TPM >1) from time-course analysis across different cell types. P-values are calculated with Wilcoxon and adjusted to FDR. Not all significant p-values are shown.
(TIF)

S10 Fig Coexpression network of genes in the three *DUX4*-detected nuclei. Twenty-three FSHD-induced genes are coexpressed (TPM >0) with *DUX4*, two of which are transcription factors, *LEUTX* and *ZSCAN4*.
(TIF)

S11 Fig RNA FISH and IF of *DUX4* and *LEUTX* in FSHD2 myotubes at days 3 and 7 of differentiation. **(A)** DUX4 RNAScope probe design. Schematic diagrams of *DUX4fl* mRNA (NM_001306068.2) and its isoform DUX4s and homologs (*DUX4C* and *DUX1)*. The "gray" sequence: almost 100% homology to *DUX4* mRNA. The "Orange" homologous sequences are different enough and would not be recognized by our *DUX4* probes. To minimize the crossdetection of *DUX4*s and *DUX4C*, we designed 6 ZZ probes (1 ZZ is a pair of RNAScope target probes): 1 ZZ falls in the region 460–1090 (common with *DUX4C*, but not in *DUX4s*), 3 ZZ in the region 1090–1418 (unique to *DUX4fl*, missing in *DUX4s* or *DUX4C*), and 2 ZZ in the region 1480–1710 (shared with *DUX4s* but missing in *DUX4C*) as indicated. Minimum 3 ZZ pairs are required for fluorescent RNAScope detection. **(B)** *LEUTX* (top) or *DUX4* (middle and bottom rows) RNAScopes are combined with immunofluorescence staining using antibody against DUX4 protein in FSHD2 myotubes at day 7 of differentiation. Myotubes containing positive *LEUTX* or *DUX4* RNA transcript signals are also positive for DUX4 protein staining. *LEUTX* or *DUX4* RNAScope signal, green; DUX4 antibody staining, red; DAPI, Blue. Yellow lines indicate the boundaries of DUX4 protein-positive myotubes. Scale bar, 10 μm. **(C)** *DUX4* (green) and *LEUTX* (red) RNAScope costaining in FSHD2-2 myotubes. DAPI is in blue. *DUX4* transcripts appear as nuclear foci (indicated with white arrows) while *LEUTX* transcripts are mostly diffuse in the cytoplasm with some additional nuclear foci. Scale bar, 10 μm.
(TIF)

S12 Fig ddSeq 3’ end RNA-seq quality metrics. **(A)** Table of number of nuclei passing each quality filter. **(B)** Mean number of reads per cell for each ddSeq replicate. **(C)** Median number of UMIs per cell for each ddSeq replicate. **(D)** Median number of genes per cell for each ddSeq replicate.
(TIF)

S13 Fig *DUX4*-detected nuclei do not exclusively cluster with nuclei with high number of FSHD-induced genes detected. **(A)** UMAP from Fig 4A split by cluster. In blue are nuclei with DUX4 detected (counts >0). Larger points indicated nuclei data from the Fluidigm. **(B)** Same as A but colored by the number of FSHD-induced genes detected (counts >0).
(TIF)

S14 Fig Comparison between published single-cell FSHD myocyte RNA-seq data [37] and single-nucleus FSHD myotube RNA-seq data in this study. **(A)** Number and percentage of *DUX4* expressing and affected myocyte single cells in published study (Supplemental table 4 of [27]) and myotube single nuclei in this study. For this study, detected is considered TPM or counts >0. **(B)** Percentage of total cells/nuclei expressing *DUX4* and 4 FSHD markers in myocyte single cells [27] and myotube single nuclei. 4 FHSD markers were selected from the published study [27] as a quality check. **(C)** Percentage of cells expressing *DUX4* (top) and percentage of DUX4-affected cells (bottom) for all FSHD or control cells for [27] and this study with 95% confidence intervals.
(TIF)

S15 Fig Gene ontology terms associated with genes upregulated in FSHD-Lo nuclei. (TIF)

S16 Fig DUX4 and DUXA binding motifs in promoters of FSHD-induced genes. **(A)** DUX4 and **(B)** DUXA binding motifs from HOCOMOCO v11. **(C)** Table of number of binding motifs for DUX4 and DUXA in the promoters of DUX4, DUXA, ZSCAN4 and LEUTX found using HOMER (Methods).
(TIF)

S17 Fig Schematic of shRNA knockdown and differentiation procedure in FSHD2-2 cells. (TIF)

S18 Fig UMAPS of ddSeq nuclei colored by expression of myogenic markers. (TIF)

S19 Fig UMAPs of ddSeq nuclei colored by expression of indicated FSHD-induced gene. ENSEMBL ID is given as well as gene name.
(TIF)

S1 Table Cell line information. Muscles biopsies were from either the tibialis anterior (TA) or the quadricep (quad). Percent methylation in D4Z4 region measured by FseI digestion.
(TIF)

S2 Table Principal component loadings for the PCAs in S3 Fig. (XLSX)

S3 Table Gene ontology for clusters from maSigPro (Fig 1B and S5 Fig). Gene ontology enrichment performed with metascape, keeping only summary terms with FDR <0.05.
(XLSX)

S4 Table Differentially expressed genes between FSHD-Hi and FSHD-Lo. Differentially expressed genes between all nuclei in FSHD-Hi and FSHD-Lo. log2FC is shown for average expression of FSHD-Hi vs FSHD-Lo. Percent of nuclei expressing the given gene in each population is indicated in Percent_FSHD-Hi and Percent_FSHD-Lo. Average expression of the gene in each population is calculated from Seurat (Methods).
(XLSX)

S5 Table Transcription factors and cofactors differentially expressed between FSHD-Hi and FSHD-Lo. (XLSX)
