# Supplementary figures and images for: Single-nucleus RNA-seq identifies divergent populations of FSHD2 myotube nuclei
Source: PLoS Genet. 2020 May 4;16(5):e1008754. doi: 10.1371/journal.pgen.1008754 (PMC7224571; doi:10.1371/journal.pgen.1008754)

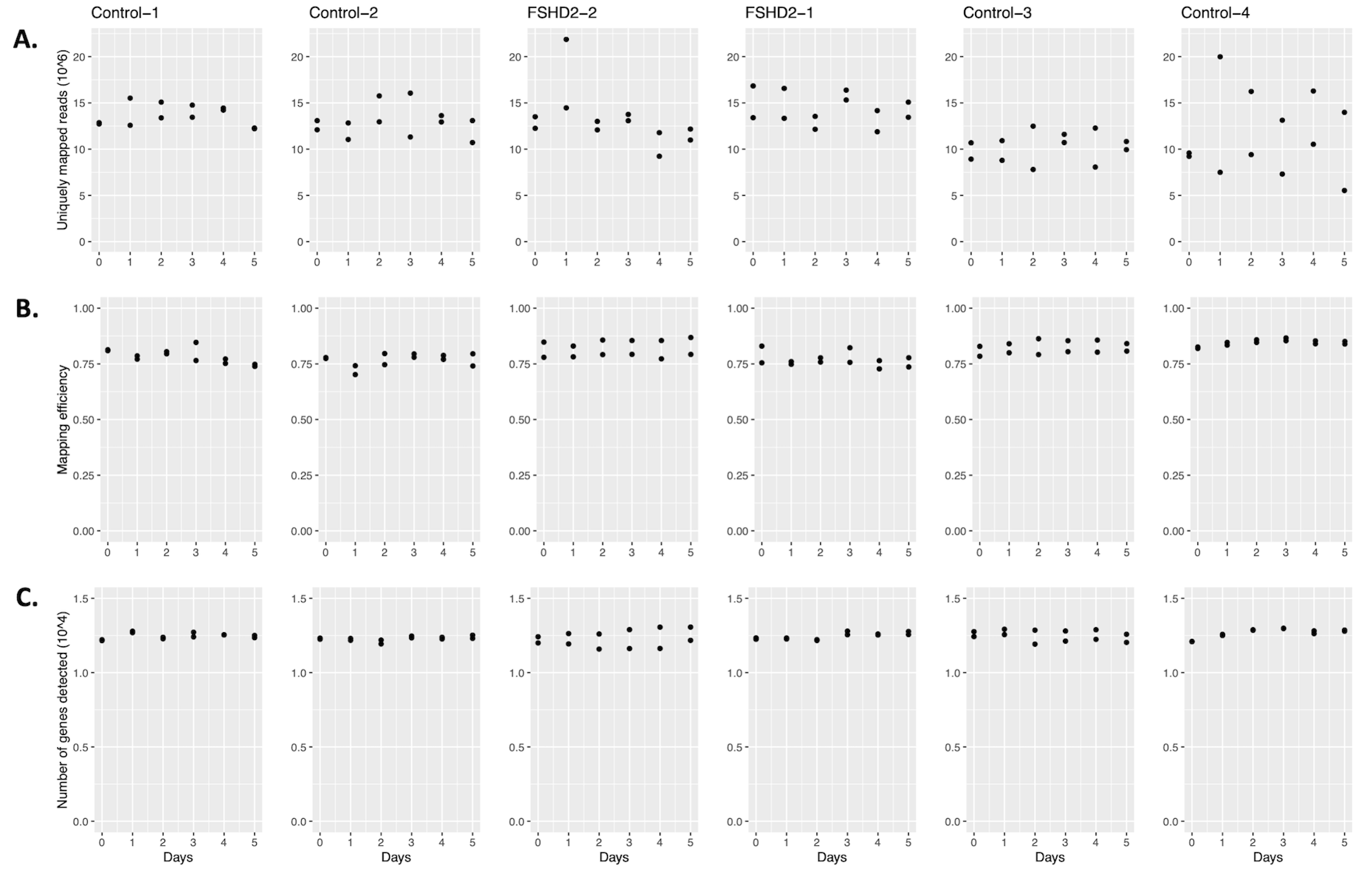

Supplement: S1 Fig — Control and FSHD2 time-course quality metrics for (A) the number of uniquely mapped reads, (B) mapping efficiency, (C) the number of genes detected (TPM> = 1). (TIF) [file pgen.1008754.s001.tif]

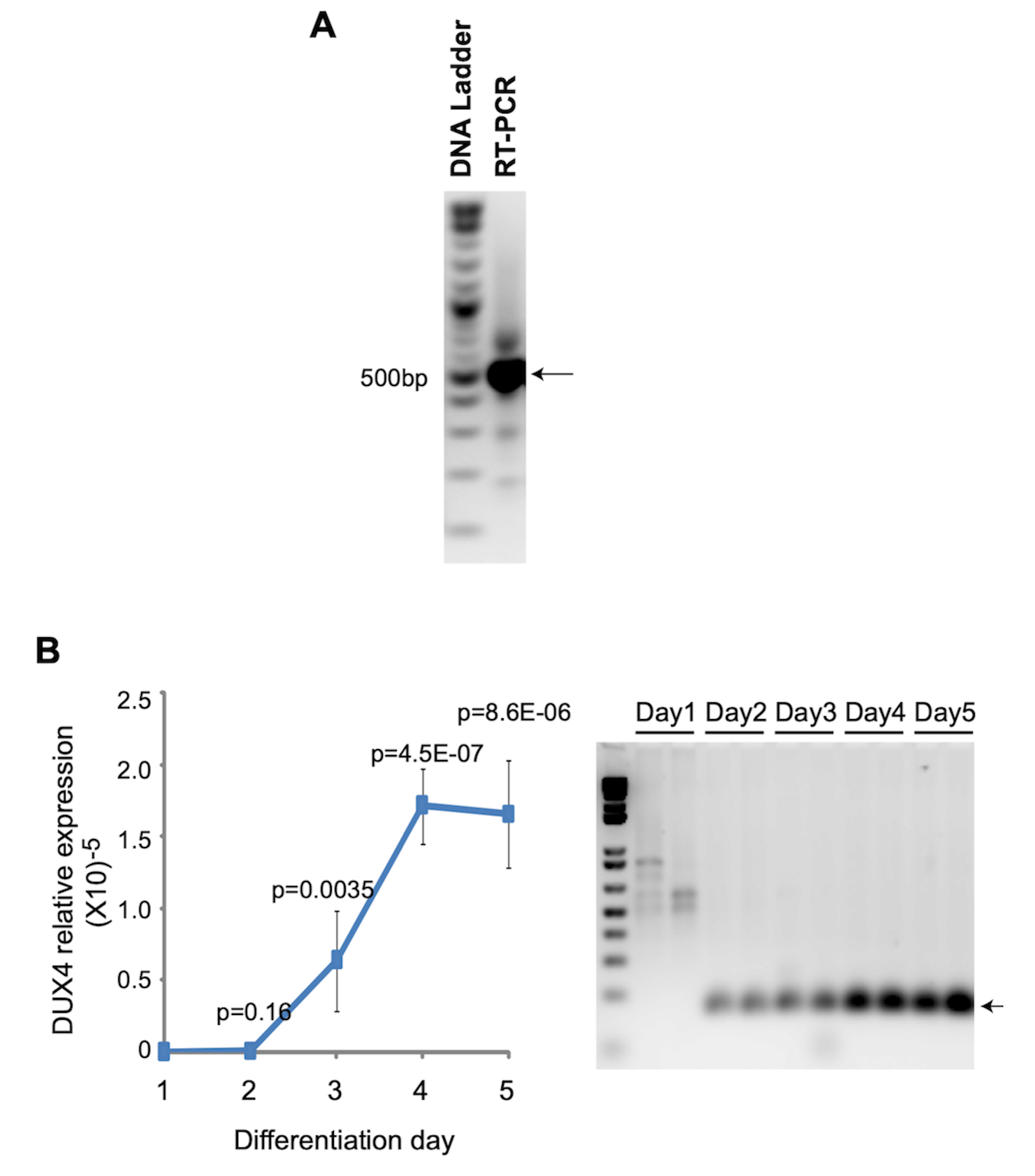

Supplement: S2 Fig — (A) Nested RT-PCR analysis of DUX4-fl expression in differentiated FSHD2-2 cells at day 3. The PCR product was sequenced to confirm its identity. The nested PCR was done using the primer sets (182–183 and 1A–184) previously published [2]. (B) FSHD2-2 cells were incubated in differentiation medium for the indicated days, and RT-qPCR was used to assess DUX4 mRNA expression during differentiation. Left, RT-qPCR data are normalized to GAPDH and the graph shows the relative abundance of DUX4 mRNA at indicated time points. Error bars are standard deviation. P values comparing to Day 1 were shown. At Day 1, the DUX4 mRNA is so low that nonspecific PCR product was amplified. Other PCR product was verified by sequencing. The qPCR primers are 5'-CCCAGGTACCAGCAGACC-3' and 5'-TCCAGGAGATGTAACTCTAATCCA-3’ [9]. Right: the qPCR products were run on the gel and their identity was confirmed by sequencing (data not show). (TIF) [file pgen.1008754.s002.tif]

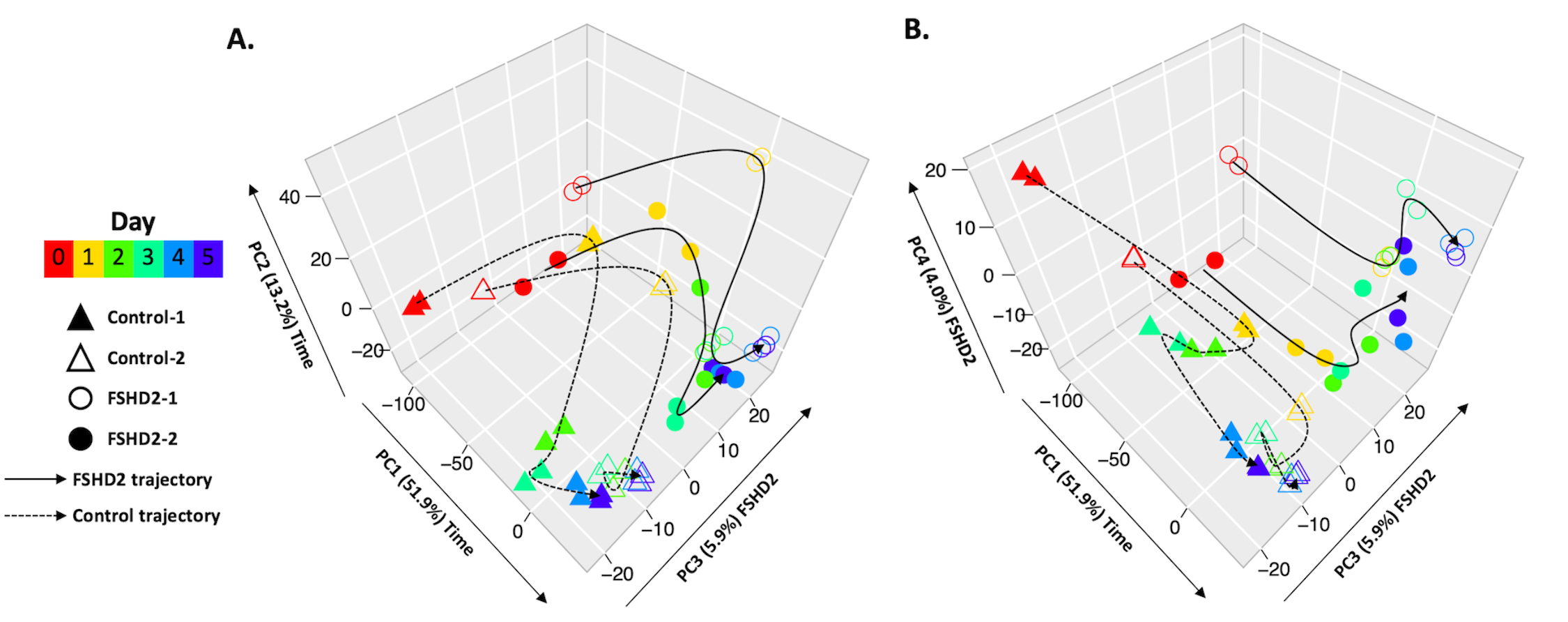

Supplement: S3 Fig — (A) PCA with PC1, PC2 and PC3 for FSHD2 and control myoblasts from tibialis anterior. PC2 further explains the expression variance across differentiation. (B) PCA with PC1, PC2, and PC3 for controls from tibialis anterior (TA) and controls from quadricep (quad). PC2 and PC3 combined explain the expression variance for muscle source and sex. Gene expression level was measured each day for duplicates by using RNA-seq. Cell types are labeled by shape, and time-points are labeled by color. (TIF) [file pgen.1008754.s003.tif]

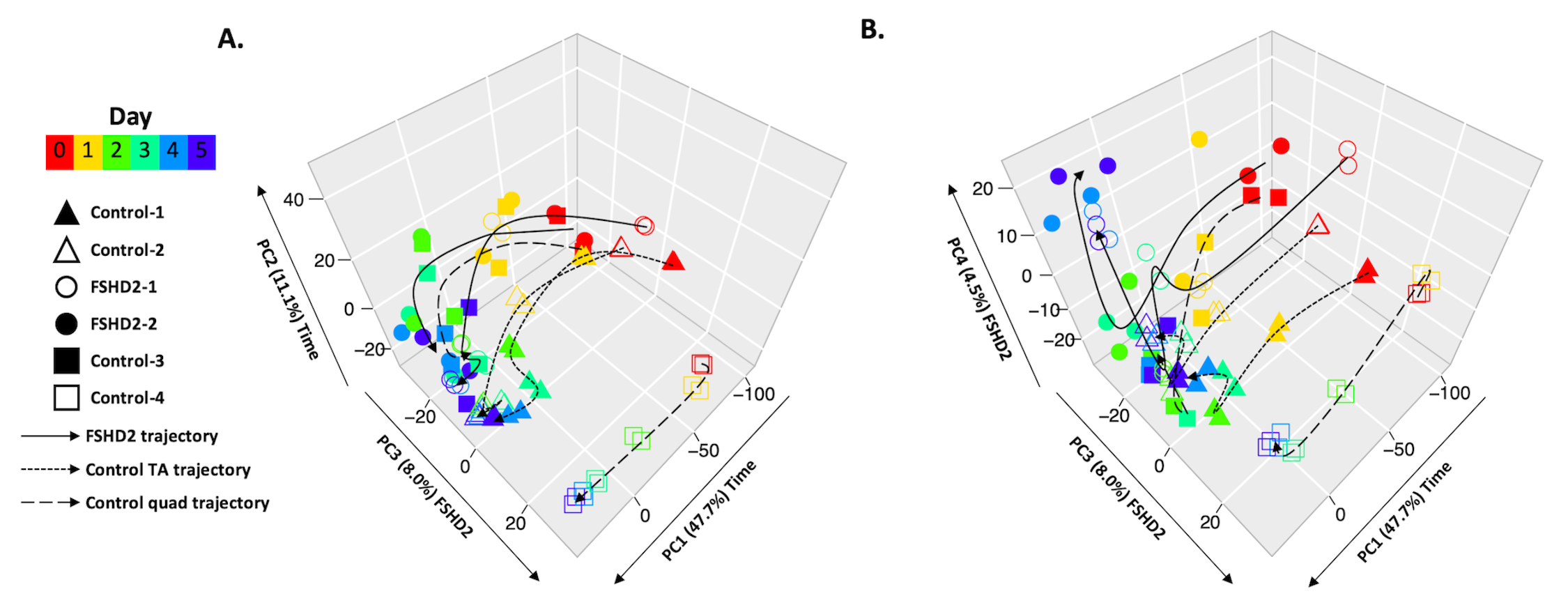

Supplement: S4 Fig — (A) PCA with PC1, PC2, and PC3 for FSHD2, controls from tibialis anterior (TA) and controls from quadricep (quad). PC2 further explains the expression variance across differentiation. (B) PCA with PC1, PC3, and PC4 for FSHD2, controls from tibialis anterior (TA) and controls from quadricep (quad). PC3 and PC4 account for variation in gene expression between FSHD2 and control samples. Gene expression level was measured each day for duplicates by using RNA-seq. Cell types are labeled by shape, and time-points are labeled by color. (TIF) [file pgen.1008754.s004.tif]

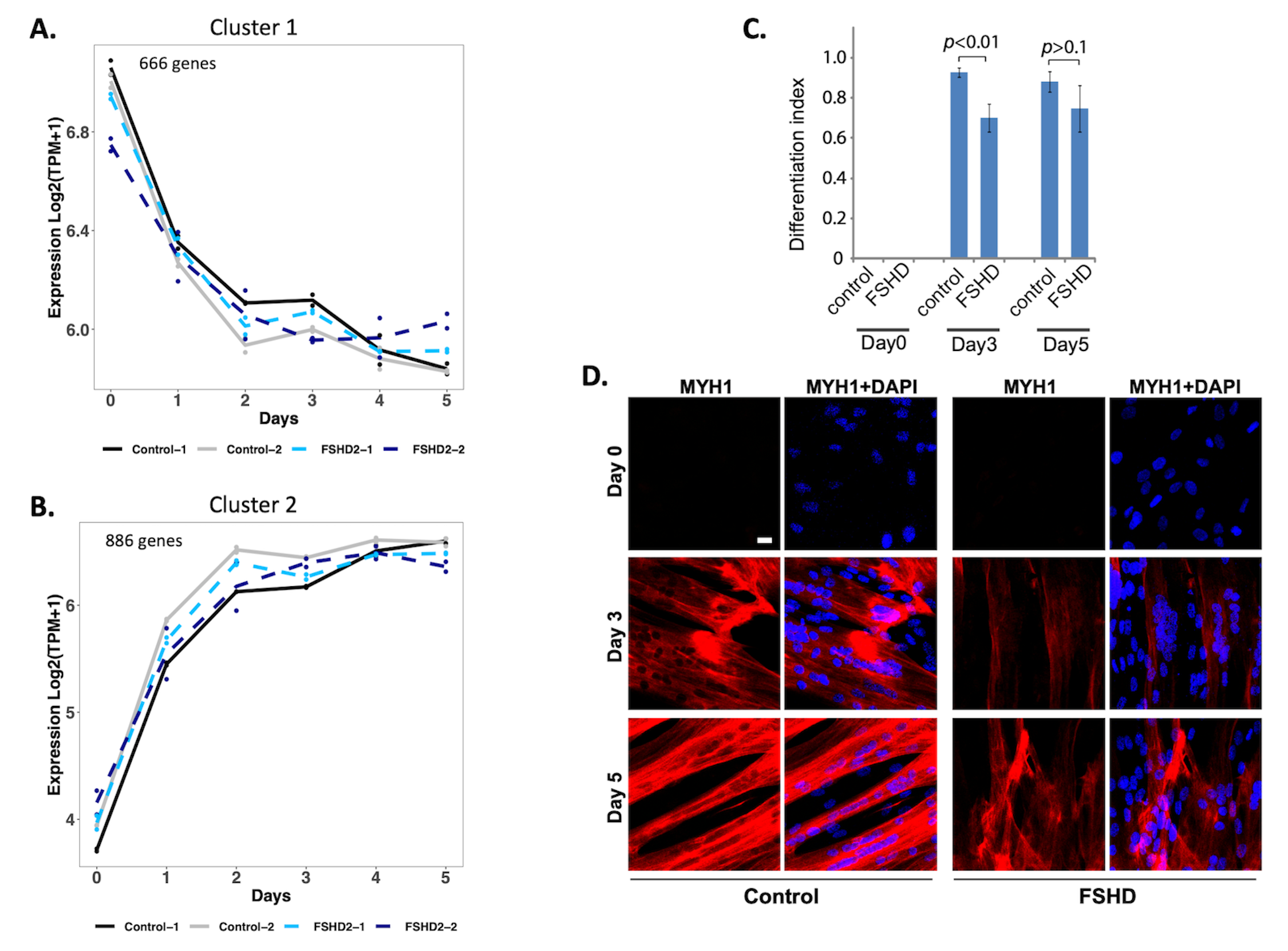

Supplement: S5 Fig — (A) Cluster 1 gene decrease during differentiation. (B) Cluster 2 gene increase during differentiation. (C) Quantification of differentiation index in myosin heavy chain1(MYH1) stained control-2 and FSHD2-2 myoblast cell lines for days 0, 3 and 5 of differentiation. Differentiation index is defined as the number of nuclei in myotubes expressing MYH1 divided by the total number of nuclei in a field. We determined the differentiation index by counting at least 600 nuclei from 3 random fields on each coverslip which was fixed at indicated days after differentiation. Myotubes with any detectable MYH1 signal are considered positive, and the signal strength of MYH1 staining is not taken into consideration. Statistically significant delay of differentiation was observed in FSHD myocytes compared to the control used on day 3 (~70% as opposed to 90%). On day 5, differentiation index is still lower in FSHD than control but the difference is no longer statistically significant. (D) Representative images of differentiation marker MYH1 (red) staining of days 0, 3 and 5 of differentiation in control-2 and FSHD2-2 cells. Bar, 10 μm. DAPI is in blue. (TIF) [file pgen.1008754.s005.tif]

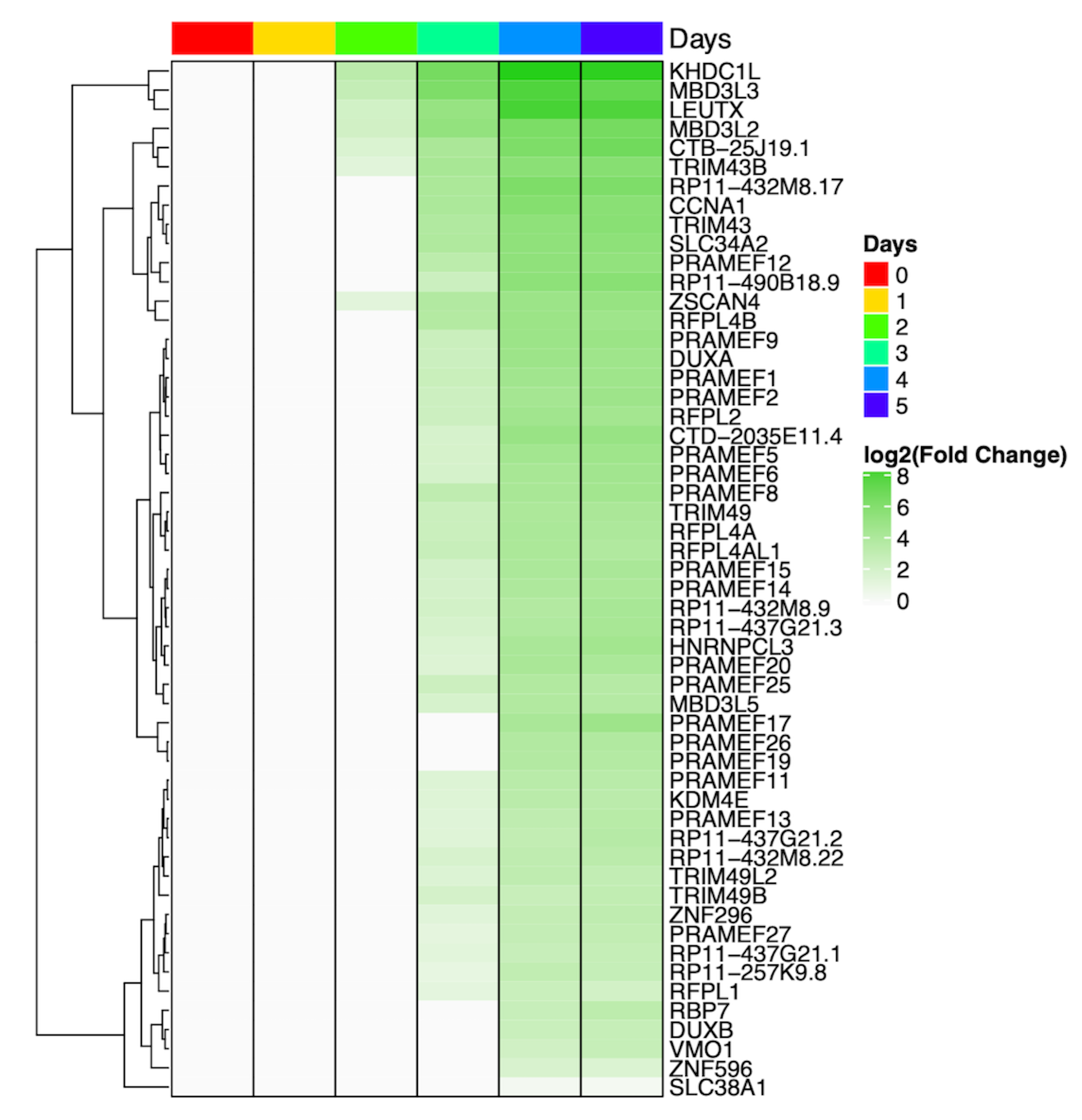

Supplement: S7 Fig — All logFC with p <0.05 are shown for comparisons of FSHD2 to control for each day of differentiation. (TIF) [file pgen.1008754.s007.tif]

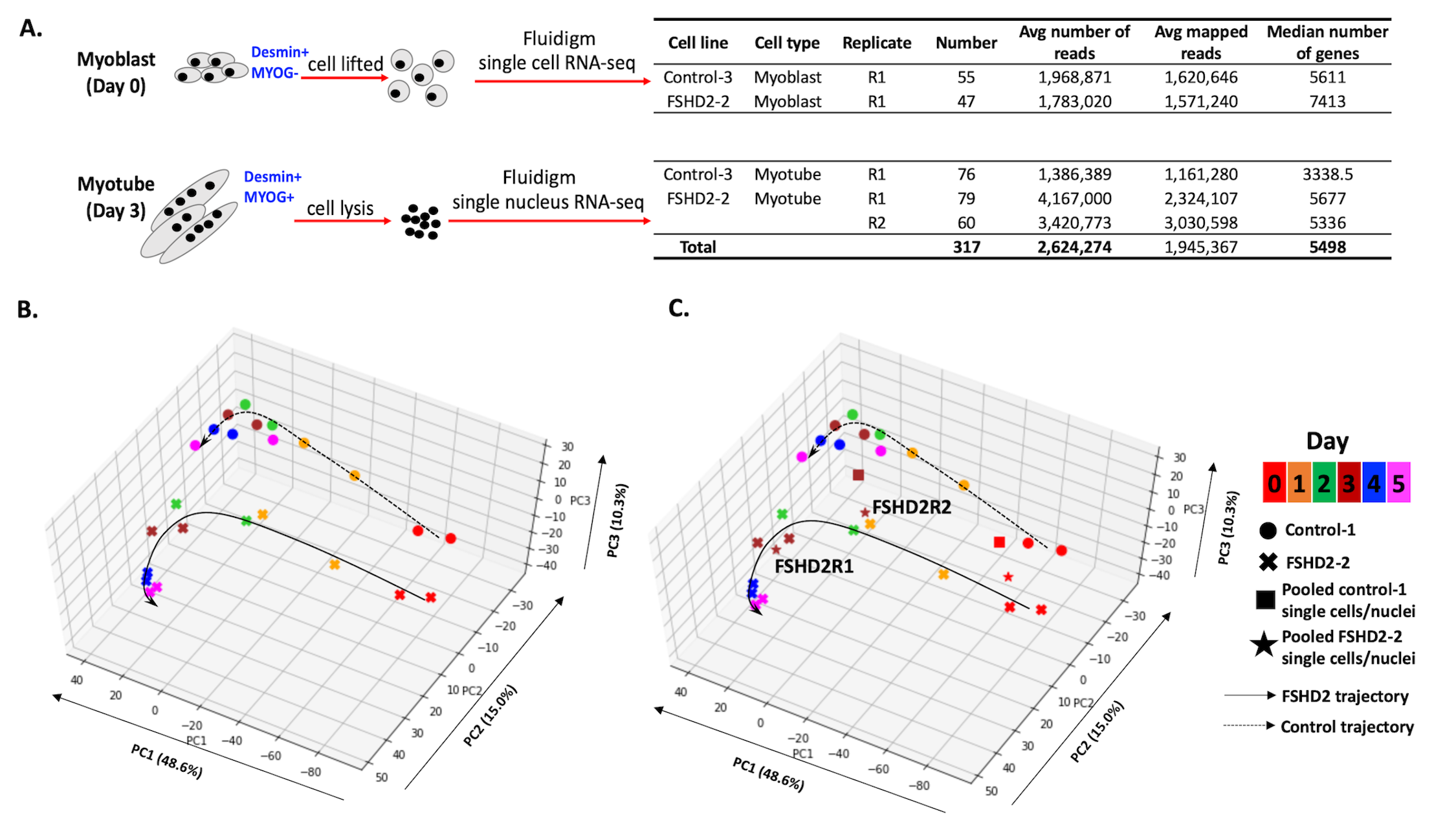

Supplement: S8 Fig — (A) Summary of single cells and single nuclei collected for sequencing. Single cells from myoblasts were selected to be desmin(+) MYOG(-) cells and retained for downstream analysis. Single nuclei from myotubes were selected to be desmin(+) MYOG(+) nuclei and retained for downstream analysis. Average number of reads, average number of mapped reads, and median number of genes detected are given per cell or nucleus for each sample. (B) Principal component analysis (PCA) of Control-1 and FSHD2-2 myoblast differentiation time-course. Gene expression level was measured each day for duplicates by using RNA-seq. Cell types are labeled by shape, and time-points are labeled by color. (C) Incremental PCA on pooled Control-1 single cells and pooled FSHD2-2 single nuclei as well as bulk Control-1 and FSHD2-2 differentiation time-courses with the same dimensions as the PCA in (B). (TIF) [file pgen.1008754.s008.tif]

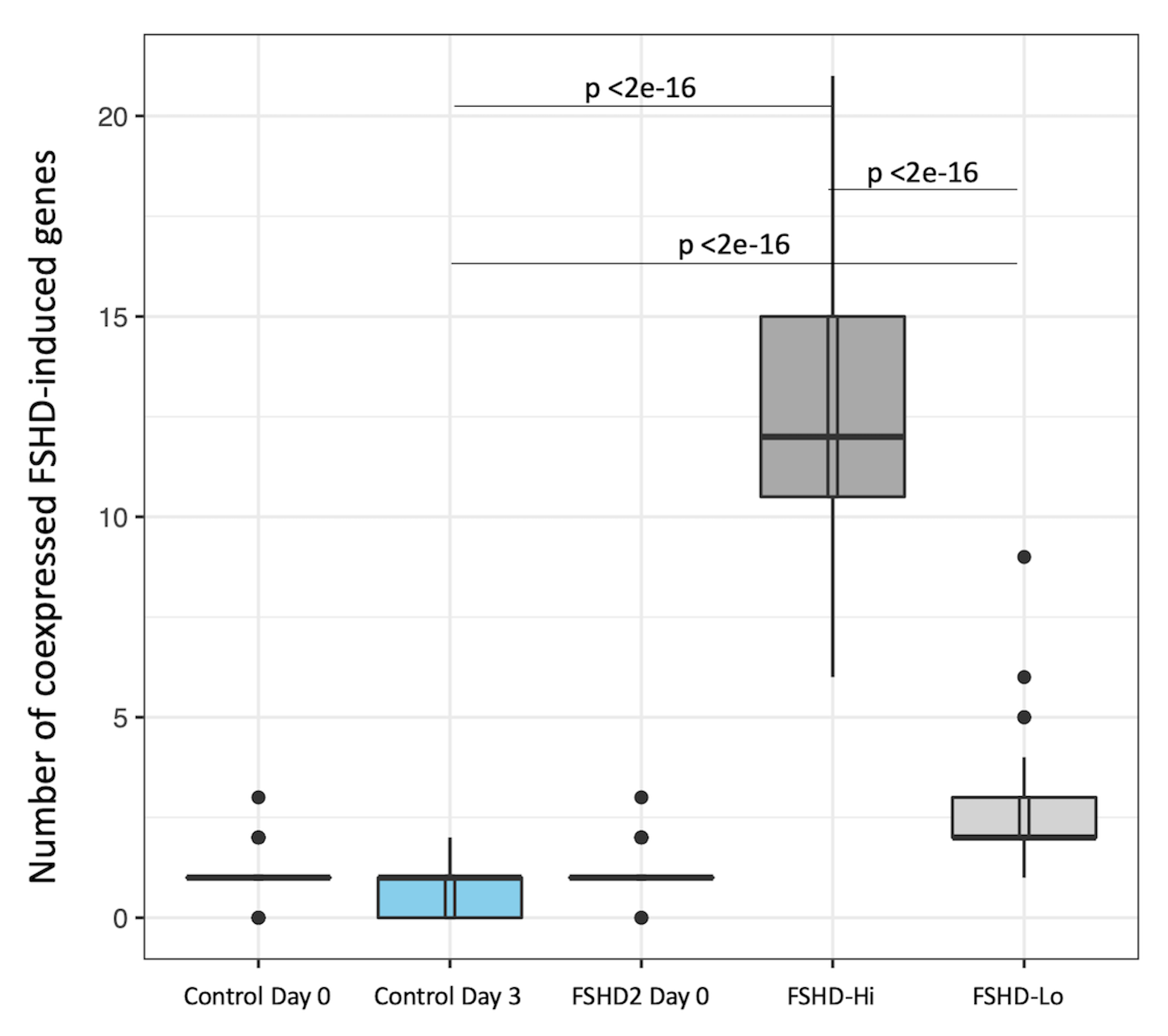

Supplement: S9 Fig — Comparison of the number of FSHD-induced genes detected (TPM >1) from time-course analysis across different cell types. P-values are calculated with Wilcoxon and adjusted to FDR. Not all significant p-values are shown. (TIF) [file pgen.1008754.s009.tif]

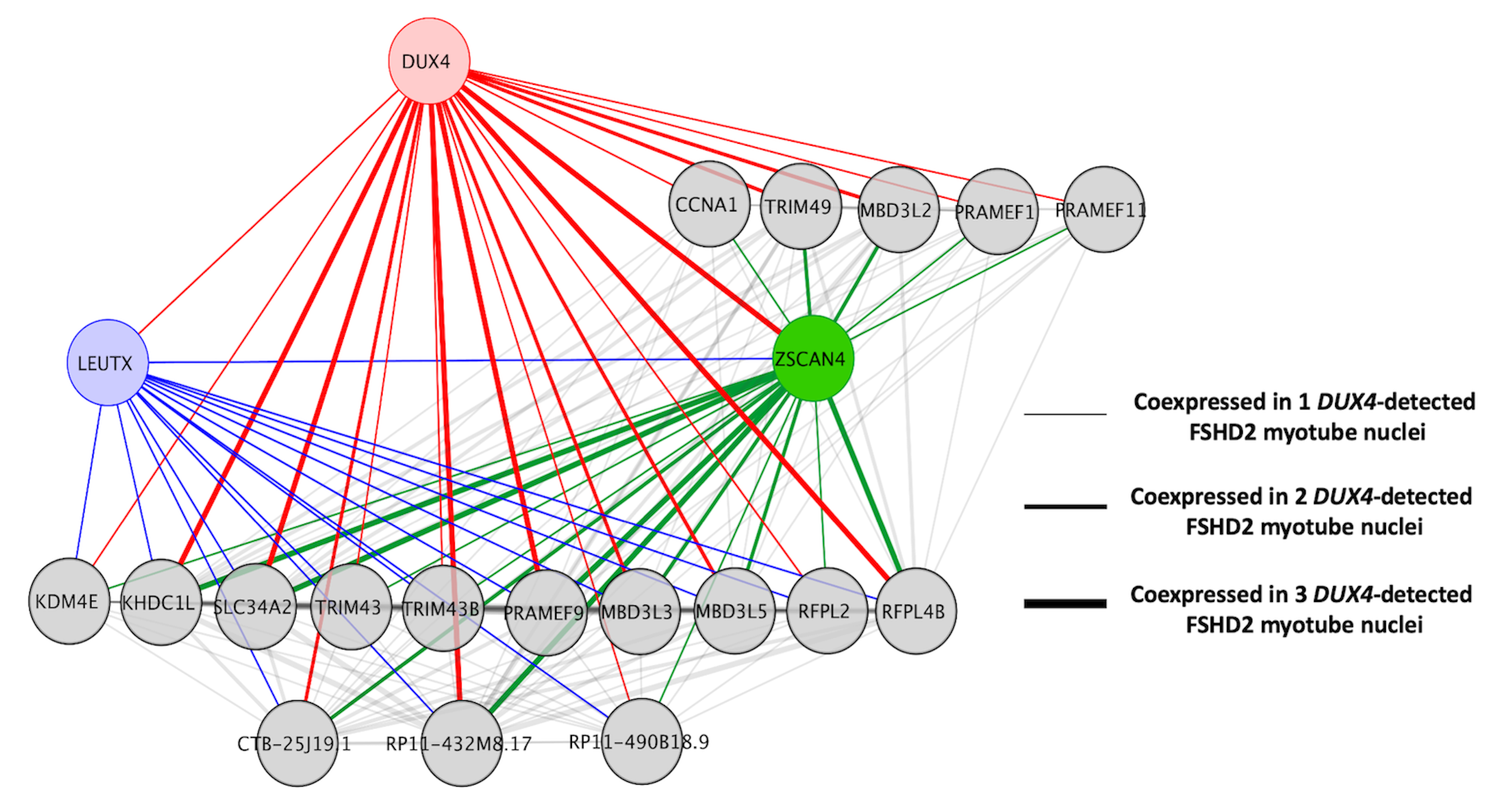

Supplement: S10 Fig — Twenty-three FSHD-induced genes are coexpressed (TPM >0) with DUX4, two of which are transcription factors, LEUTX and ZSCAN4. (TIF) [file pgen.1008754.s010.tif]

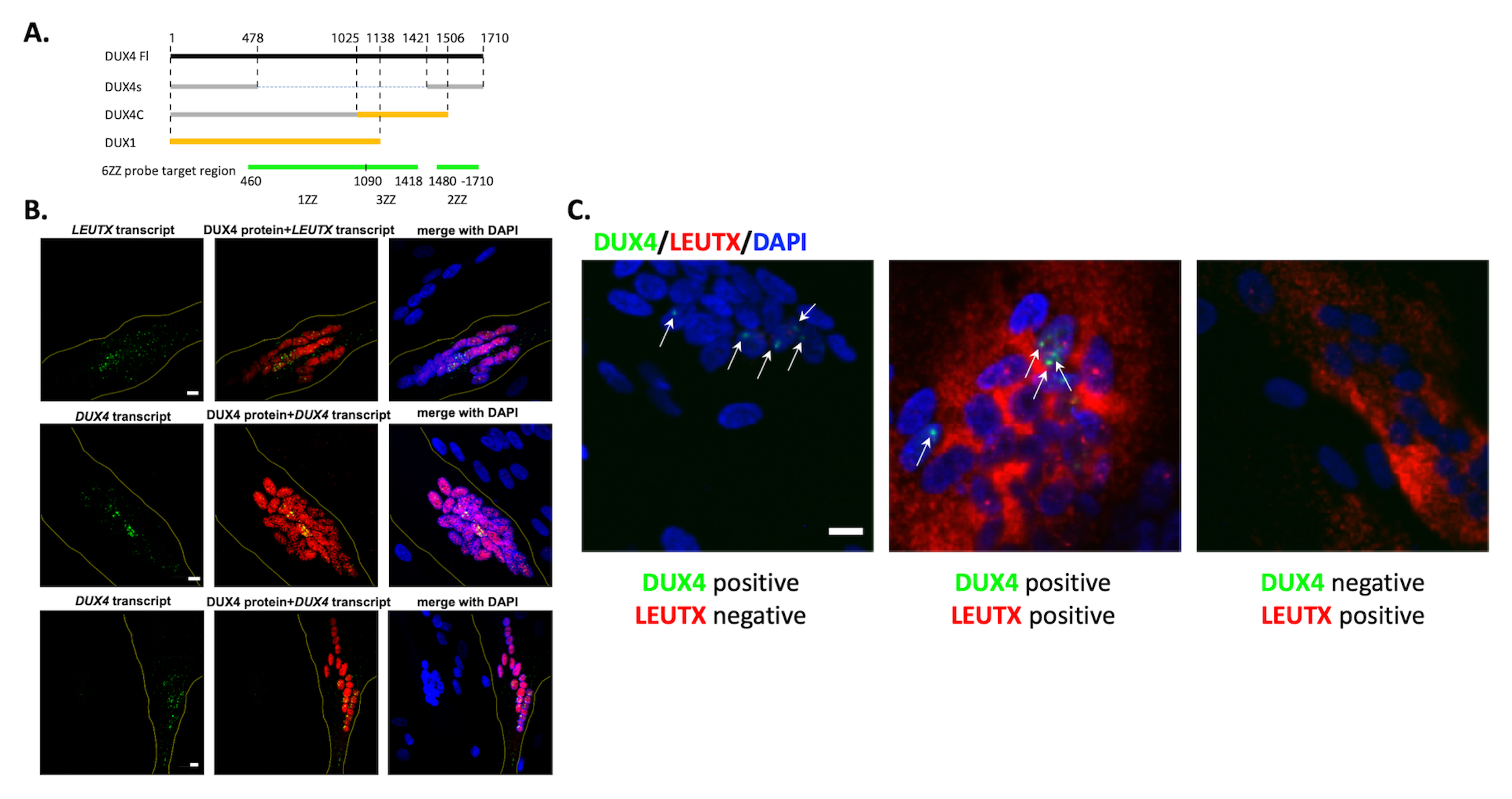

Supplement: S11 Fig — (A) DUX4 RNAScope probe design. Schematic diagrams of DUX4fl mRNA (NM_001306068.2) and its isoform DUX4s and homologs (DUX4C and DUX1). The "gray" sequence: almost 100% homology to DUX4 mRNA. The "Orange" homologous sequences are different enough and would not be recognized by our DUX4 probes. To minimize the crossdetection of DUX4s and DUX4C, we designed 6 ZZ probes (1 ZZ is a pair of RNAScope target probes): 1 ZZ falls in the region 460–1090 (common with DUX4C, but not in DUX4s), 3 ZZ in the region 1090–1418 (unique to DUX4fl, missing in DUX4s or DUX4C), and 2 ZZ in the region 1480–1710 (shared with DUX4s but missing in DUX4C) as indicated. Minimum 3 ZZ pairs are required for fluorescent RNAScope detection. (B) LEUTX (top) or DUX4 (middle and bottom rows) RNAScopes are combined with immunofluorescence staining using antibody against DUX4 protein in FSHD2 myotubes at day 7 of differentiation. Myotubes containing positive LEUTX or DUX4 RNA transcript signals are also positive for DUX4 protein staining. LEUTX or DUX4 RNAScope signal, green; DUX4 antibody staining, red; DAPI, Blue. Yellow lines indicate the boundaries of DUX4 protein-positive myotubes. Scale bar, 10 μm. (C) DUX4 (green) and LEUTX (red) RNAScope costaining in FSHD2-2 myotubes. DAPI is in blue. DUX4 transcripts appear as nuclear foci (indicated with white arrows) while LEUTX transcripts are mostly diffuse in the cytoplasm with some additional nuclear foci. Scale bar, 10 μm. (TIF) [file pgen.1008754.s011.tif]

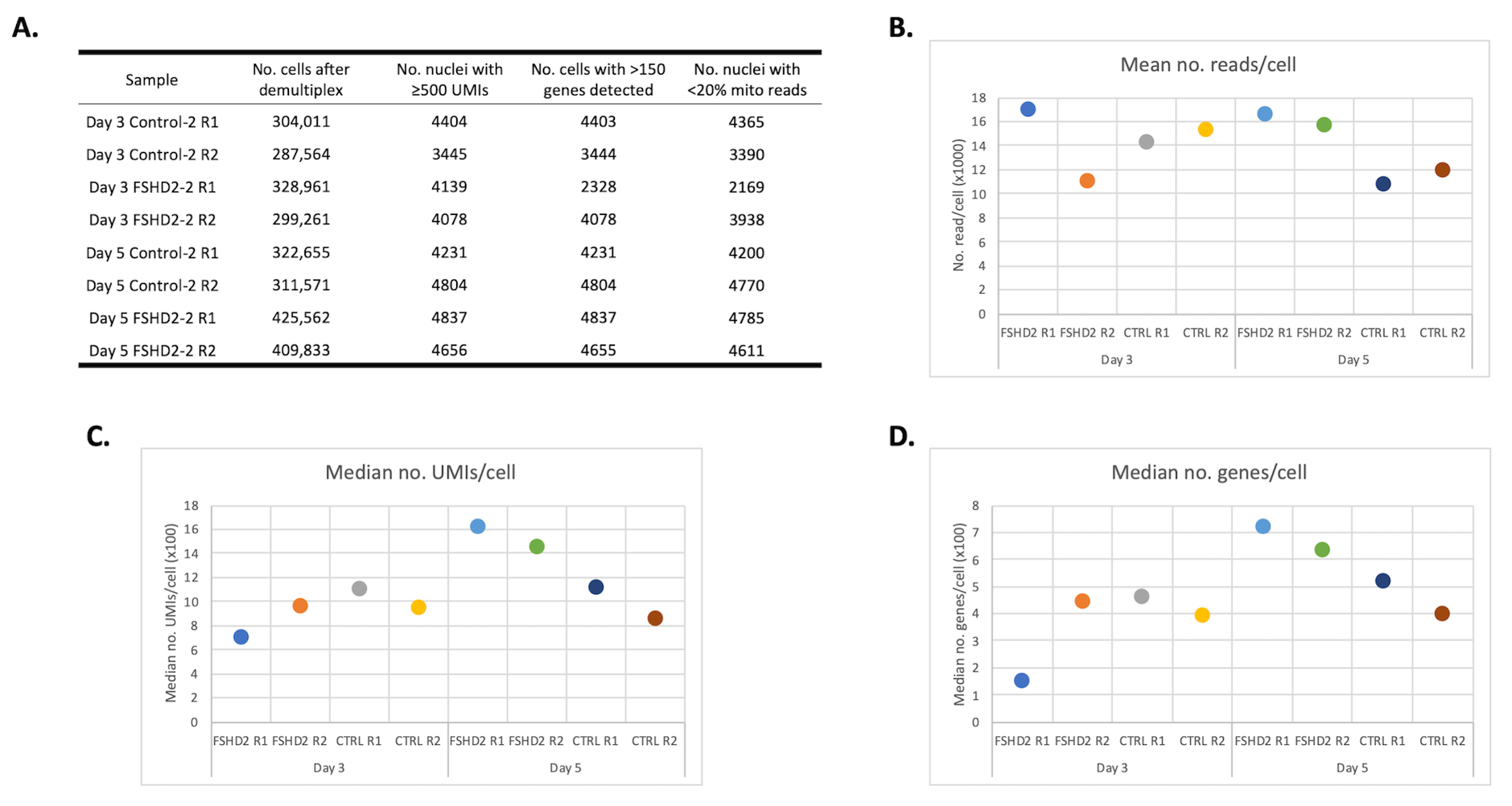

Supplement: S12 Fig — (A) Table of number of nuclei passing each quality filter. (B) Mean number of reads per cell for each ddSeq replicate. (C) Median number of UMIs per cell for each ddSeq replicate. (D) Median number of genes per cell for each ddSeq replicate. (TIF) [file pgen.1008754.s012.tif]

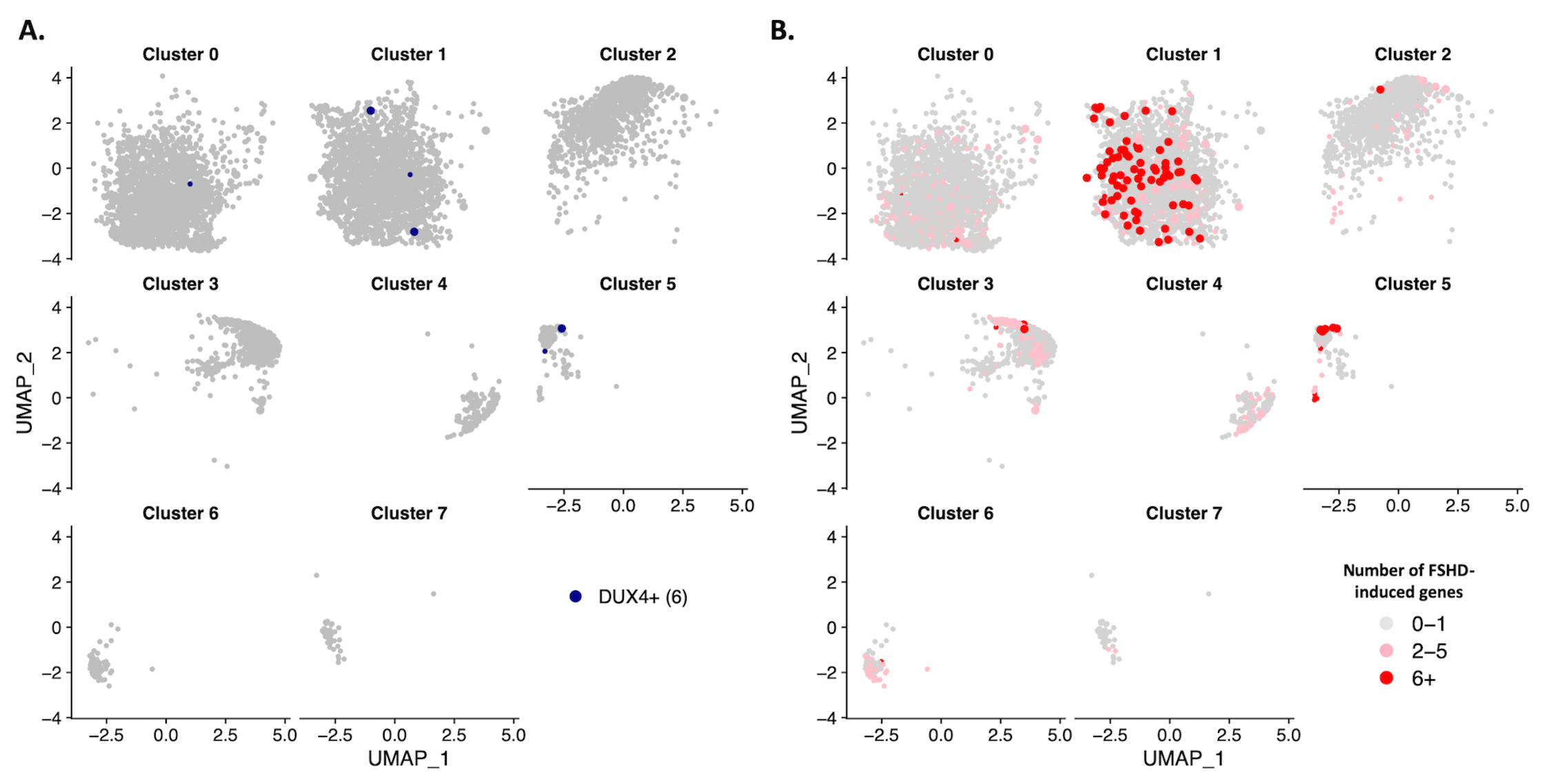

Supplement: S13 Fig — (A) UMAP from Fig 4A split by cluster. In blue are nuclei with DUX4 detected (counts >0). Larger points indicated nuclei data from the Fluidigm. (B) Same as A but colored by the number of FSHD-induced genes detected (counts >0). (TIF) [file pgen.1008754.s013.tif]

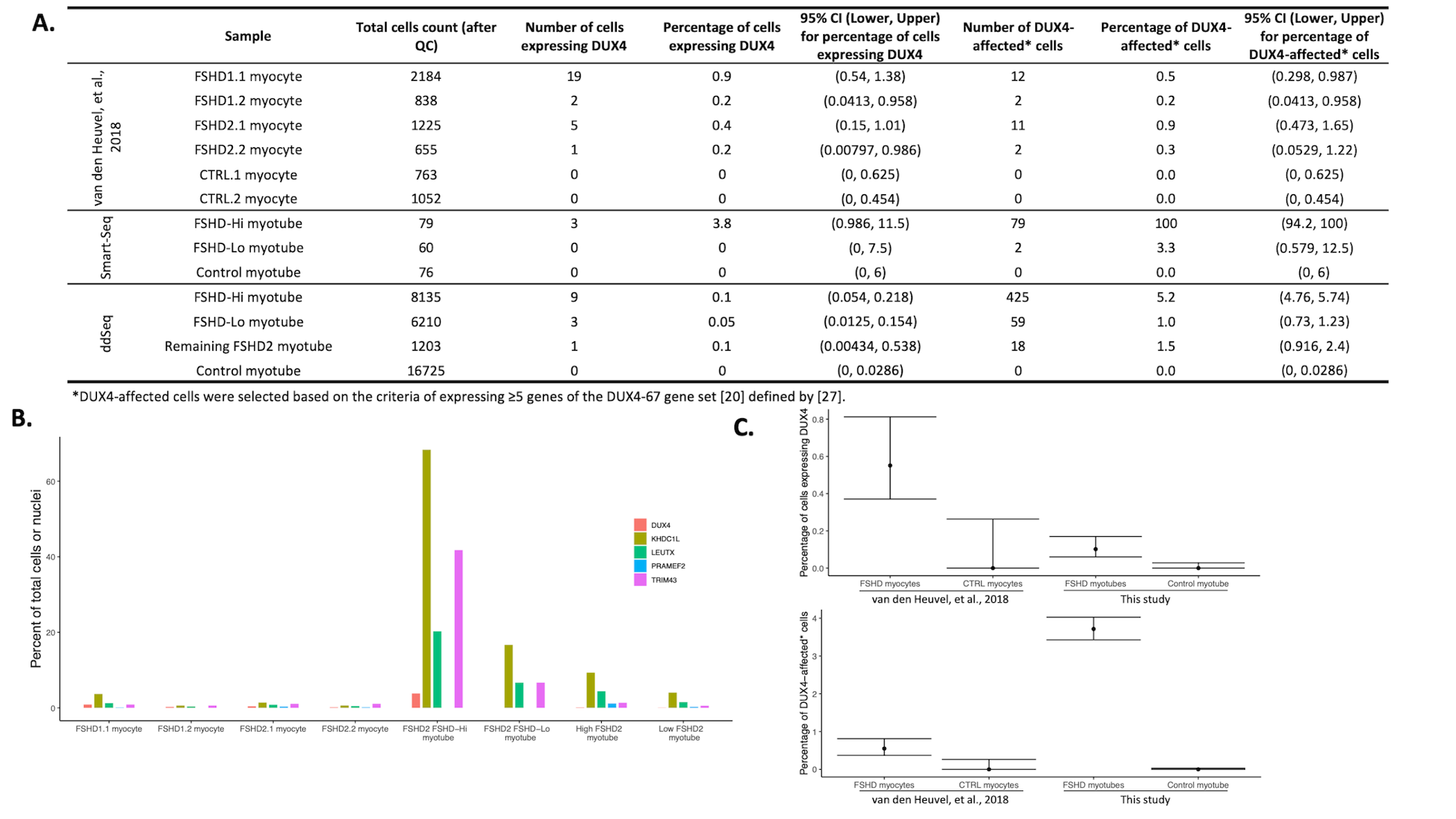

Supplement: S14 Fig — (A) Number and percentage of DUX4 expressing and affected myocyte single cells in published study (Supplemental table 4 of [27]) and myotube single nuclei in this study. For this study, detected is considered TPM or counts >0. (B) Percentage of total cells/nuclei expressing DUX4 and 4 FSHD markers in myocyte single cells [27] and myotube single nuclei. 4 FHSD markers were selected from the published study [27] as a quality check. (C) Percentage of cells expressing DUX4 (top) and percentage of DUX4-affected cells (bottom) for all FSHD or control cells for [27] and this study with 95% confidence intervals. (TIF) [file pgen.1008754.s014.tif]

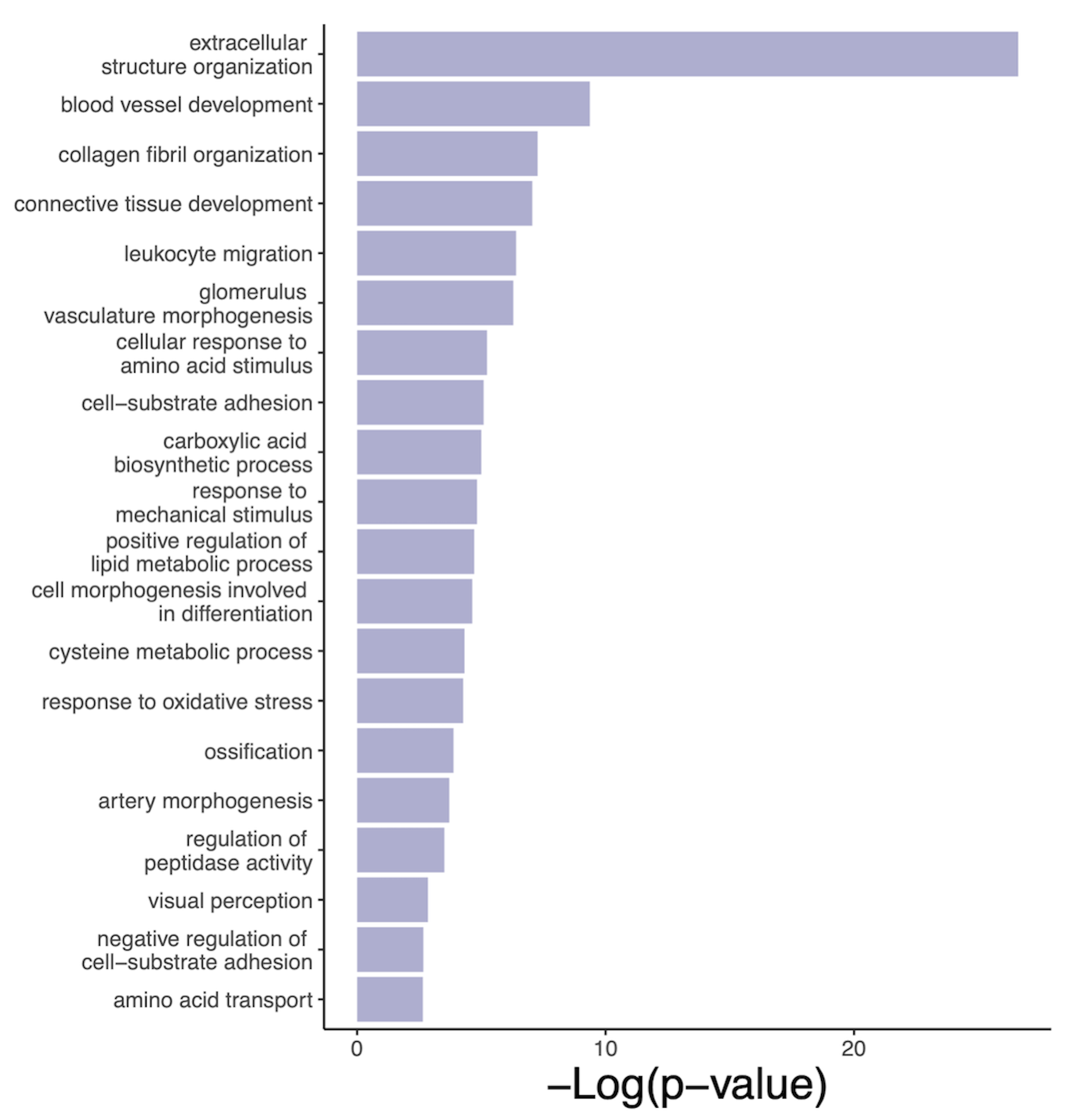

Supplement: S15 Fig — (TIF) [file pgen.1008754.s015.tif]

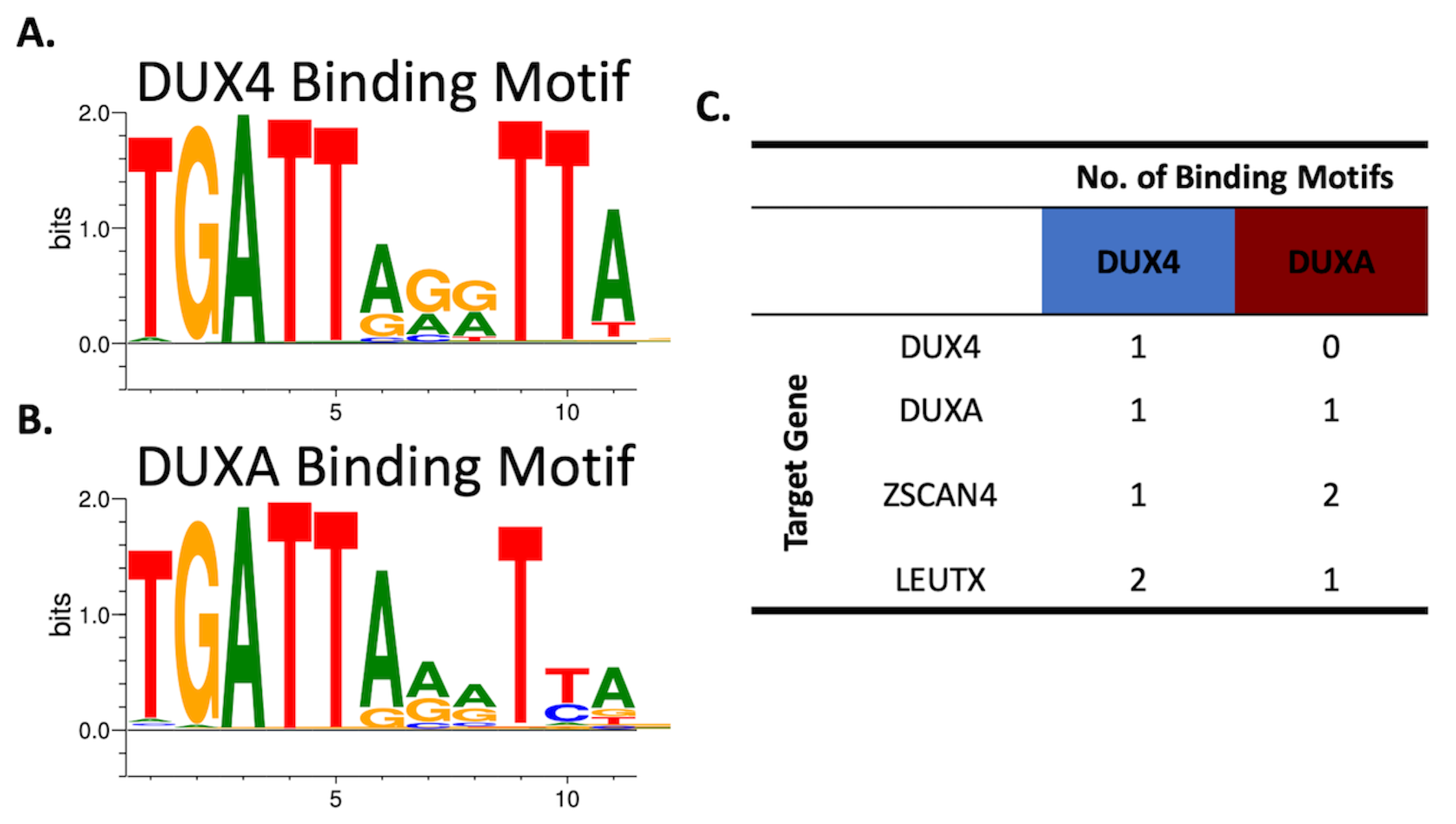

Supplement: S16 Fig — (A) DUX4 and (B) DUXA binding motifs from HOCOMOCO v11. (C) Table of number of binding motifs for DUX4 and DUXA in the promoters of DUX4, DUXA, ZSCAN4 and LEUTX found using HOMER (Methods). (TIF) [file pgen.1008754.s016.tif]

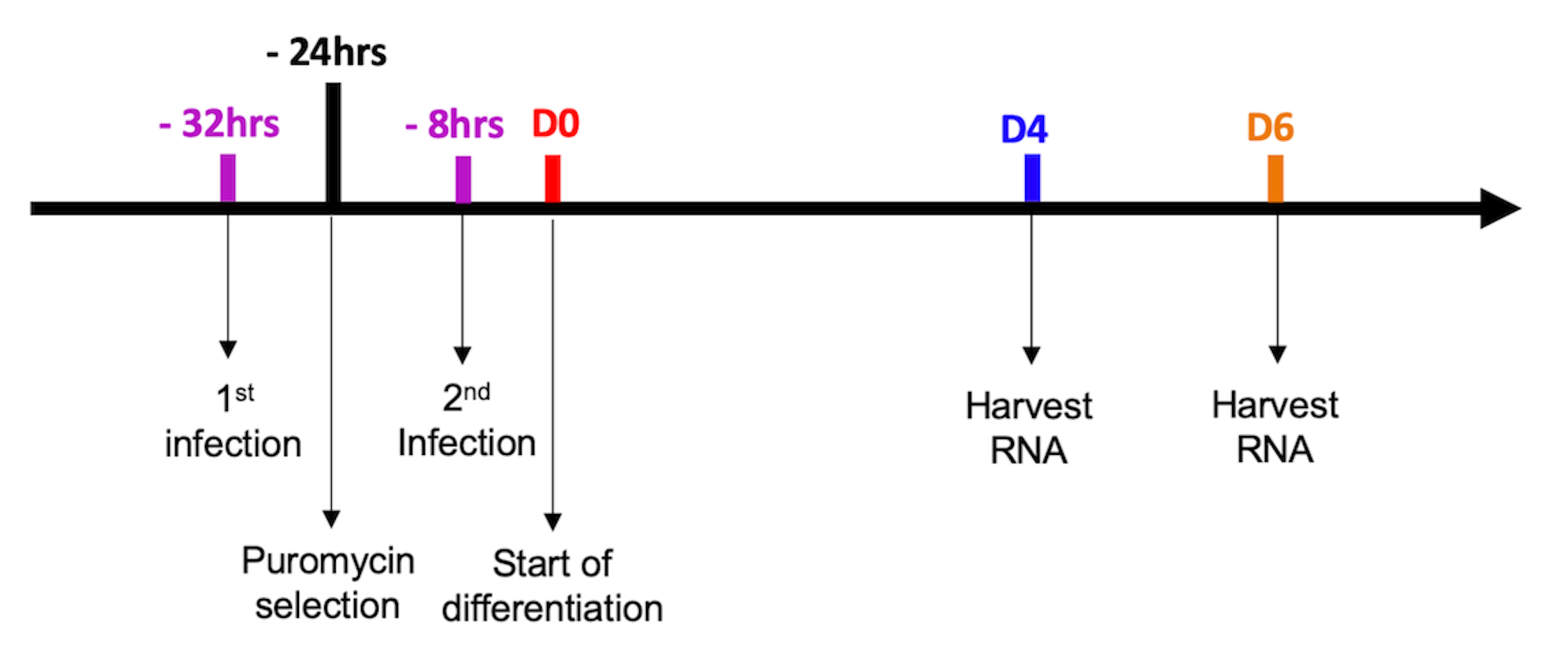

Supplement: S17 Fig — (TIF) [file pgen.1008754.s017.tif]

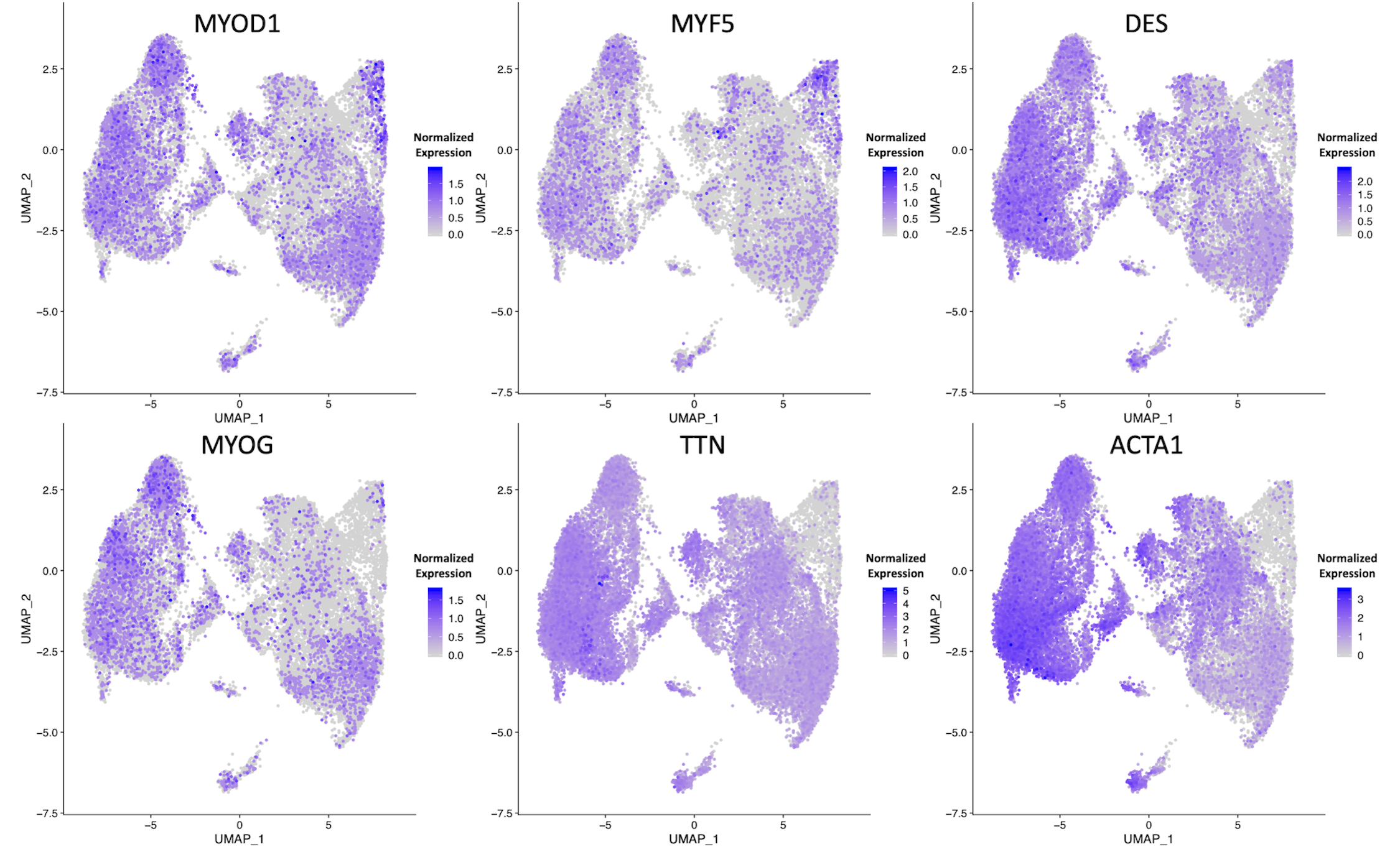

Supplement: S18 Fig — (TIF) [file pgen.1008754.s018.tif]

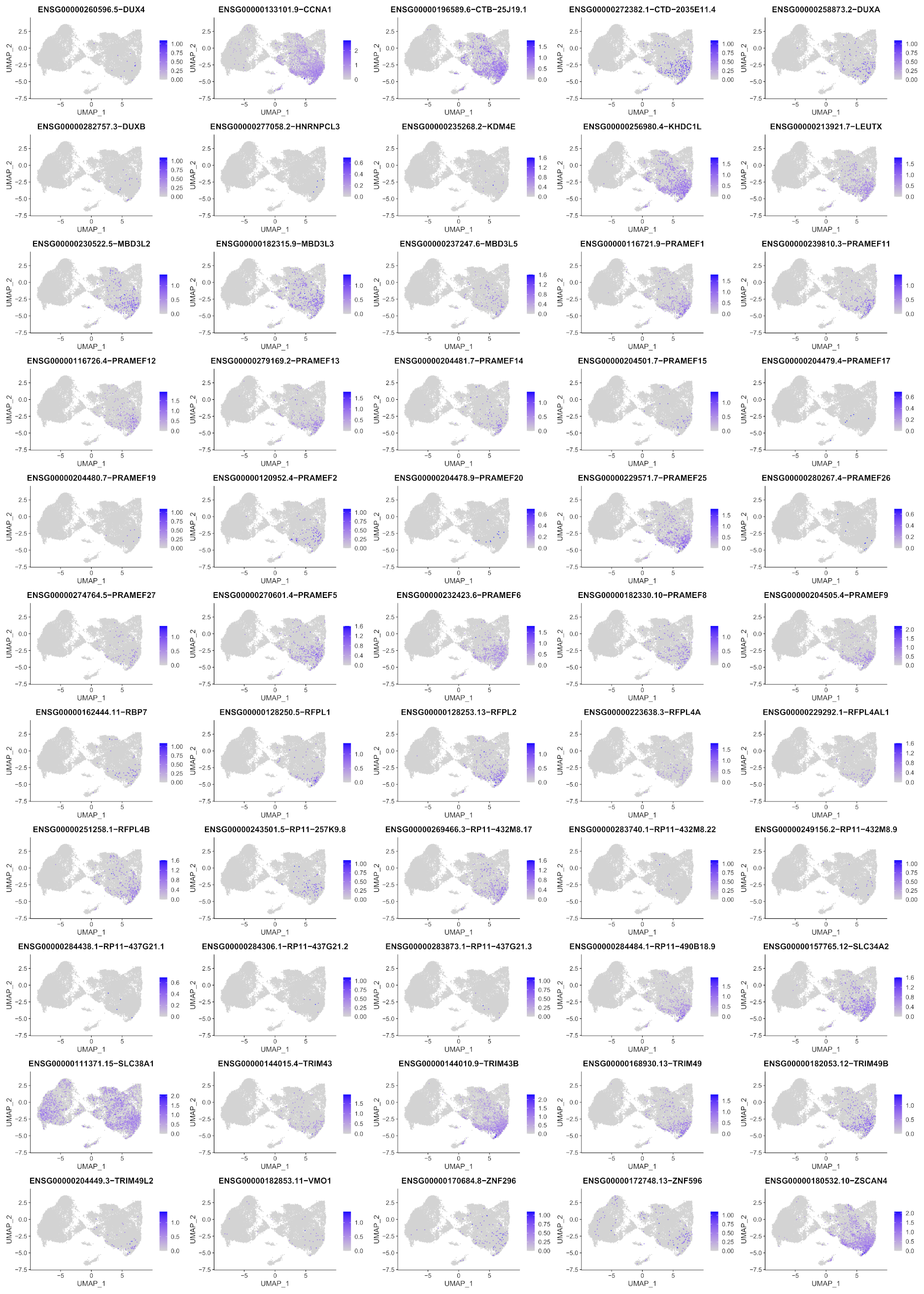

Supplement: S19 Fig — ENSEMBL ID is given as well as gene name. (TIF) [file pgen.1008754.s019.tif]

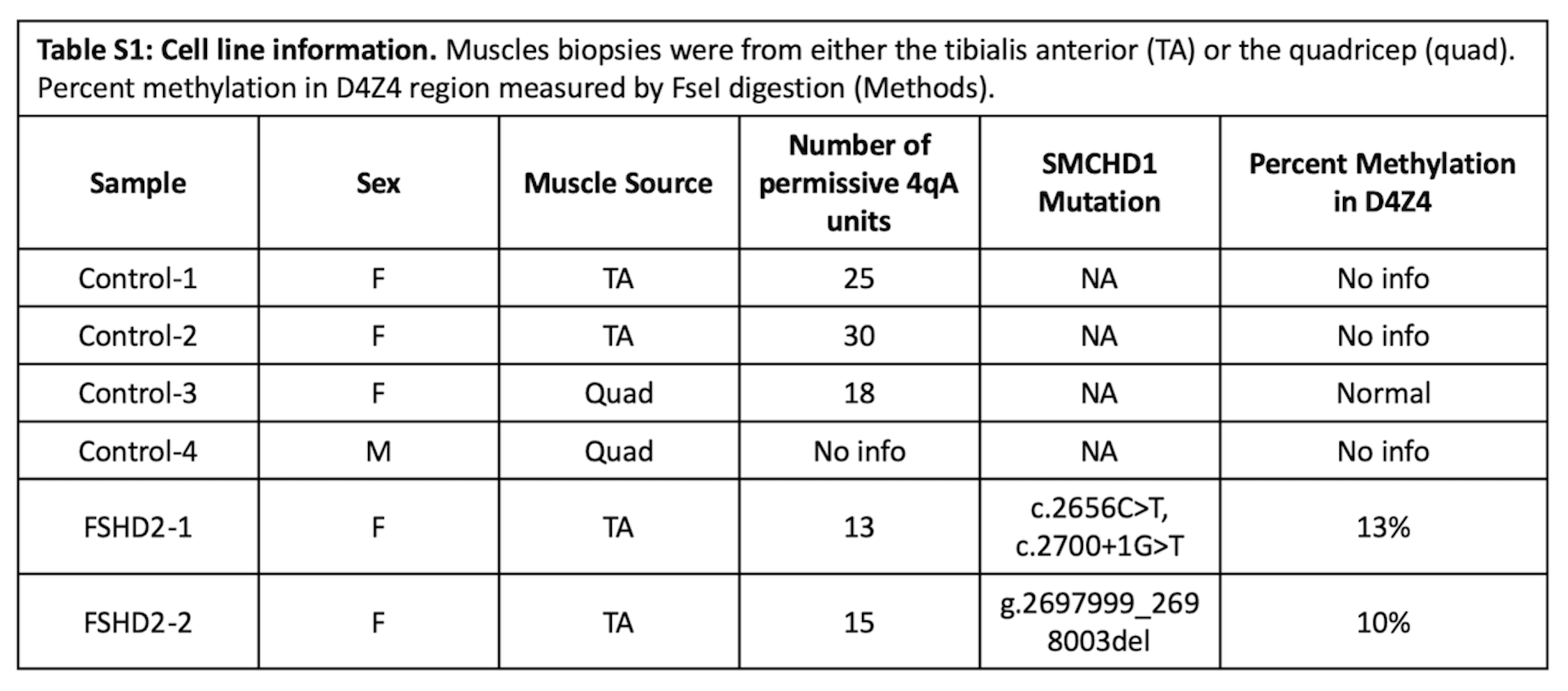

Supplement: S1 Table — Muscles biopsies were from either the tibialis anterior (TA) or the quadricep (quad). Percent methylation in D4Z4 region measured by FseI digestion. (TIF) [file pgen.1008754.s020.tif]
